# Considering developmental neurotoxicity *in vitro* data for human health risk assessment using physiologically-based kinetic modeling: deltamethrin case study

**DOI:** 10.1093/toxsci/kfad007

**Published:** 2023-01-13

**Authors:** Christian Maass, Stephan Schaller, André Dallmann, Kathrin Bothe, Dennis Müller

**Affiliations:** esqLABS GmbH, Hambierich 34, Saterland 26683, Germany; esqLABS GmbH, Hambierich 34, Saterland 26683, Germany; Pharmacometrics/Modeling and Simulation, Research and Development, Pharmaceuticals, Bayer AG, Leverkusen 51373, Germany; Regulatory Toxicology, Research and Development, Bayer AG, CropScience, 40789 Monheim am Rhein, Germany; Regulatory Toxicology, Research and Development, Bayer AG, CropScience, 40789 Monheim am Rhein, Germany

**Keywords:** modeling and simulation, physiologically based kinetics (PBK), new-approach methodologies (NAMs), fetal brain exposure, deltamethrin, developmental neurotoxicity (DNT)

## Abstract

Developmental neurotoxicity (DNT) is a potential hazard of chemicals. Recently, an *in vitro* testing battery (DNT IVB) was established to complement existing rodent *in vivo* approaches. Deltamethrin (DLT), a pyrethroid with a well-characterized neurotoxic mode of action, has been selected as a reference chemical to evaluate the performance of the DNT IVB. The present study provides context for evaluating the relevance of these DNT IVB results for the human health risk assessment of DLT by estimating potential human fetal brain concentrations after maternal exposure to DLT. We developed a physiologically based kinetic (PBK) model for rats which was then translated to humans considering realistic *in vivo* exposure conditions (acceptable daily intake [ADI] for DLT). To address existing uncertainties, we designed case studies considering the most relevant drivers of DLT uptake and distribution. Calculated human fetal brain concentrations were then compared with the lowest benchmark concentration achieved in the DNT IVB. The developed rat PBK model was validated on *in vivo* rat toxicokinetic data of DLT over a broad range of doses. The uncertainty based case study evaluation confirmed that repeated exposure to DLT at an ADI level would likely result in human fetal brain concentrations far below the *in vitro* benchmark. The presented results indicate that DLT concentrations in the human fetal brain are highly unlikely to reach concentrations associated with *in vitro* findings under realistic exposure conditions. Therefore, the new *in vitro* DNT results are considered to have no impact on the current risk assessment approach.

##  

Developmental neurotoxicity (DNT) is a potential hazard of chemicals related to an adverse effect on the normal development of nervous system structure or function. Experiments with rats according to OECD test guidelines (TG) 426 (DNT study) and TG 443 (extended 1-generation reproductive toxicity study) (OECD, 2007, 2018) are up to now considered the “gold standard” for DNT hazard identification and risk assessment. However, the sensitivity and predictivity for the protection of the human developing brain are limited by species differences in exposure, developmental timing (eg, pre vs postnatal), and pharmaco-/toxicodynamic factors (Dorman *et al.*, 2001; Tsuji and Crofton, 2012). In addition, time and resource implications, and the need to limit animal use, resulted in a consensus of scientific stakeholders from regulatory agencies, academia, and industry that a new framework for DNT assessment of chemicals based on a battery of *in vitro* assays (DNT IVB) is needed. Deltamethrin (DLT) was chosen as one of the case study (ie, reference) compounds to assess the suitability of the new DNT IVB for regulatory decision-making, due to its well-characterized neurotoxic mode of action and availability of kinetic data (EFSA PPR Panel *et al.*, 2021; Masjosthusmann *et al.*, 2020).

DLT is a pyrethroid and used as an insecticide in plant protection and biocide products. Based on an available DNT *in vivo* study conducted according to OECD 426 (Bayer, 2011), existing regulatory assessments concluded that DLT is not a developmental neurotoxicant. However, *in vitro* studies have shown an impact on neuronal network formation in rat and human cells (r/hNNF), an effect on oligodendrocyte differentiation in human neural progenitor cells (NPC5), and an effect on migration in human neural crest cells (UKN2) within the new DNT IVB. The effect in the rNNF (benchmark concentration 50 [BMC] 0.5 µM) and NPC5 (BMC30 0.6 µM) was the most sensitive endpoints (Masjosthusmann *et al.*, 2020). As these findings indicate a potential DNT hazard DLT might exert on the neuronal development, it is important to investigate the potential impact on the current risk assessment for DLT, which is largely based on animal *in vivo* data. In chemical risk assessment, nonanimal or new approach methodologies (NAMs), including *in vitro* models and physiologically based kinetic (PBK) models, have emerged as a tool used in several sectors (pharmaceuticals, veterinary medicine, cosmetics, and agrochemicals) and are considered to play a pivotal role for next-generation risk assessment (NGRA) (Pallocca *et al.*, 2022). As NAMs are being more and more refined, the role of PBK modeling is key when it comes to the integration, interpretation, extrapolation, and translation of data from multiple scales of biology.

Recently, PBK models of DLT and other pyrethroids in rats and humans have been developed and used to investigate age-specific differences in kinetics and internal target tissue exposure (eg, in adult brain) (Godin *et al.*, 2010; Mallick *et al.*, 2020; Personne *et al.*, 2021). However, these models did not include a fetal compartment, which is essential for the model to estimate the potential DLT concentrations in the fetal brain under defined circumstances of exposure or were not developed in humans.

Therefore, in the present study, PBK modeling was utilized to put these new *in vitro* results into context by providing estimations for human fetal brain concentrations under realistic “worst-case” *in vivo* exposure conditions (0.01 mg/kg bodyweight [bw]/day, acceptable daily intake [ADI] for DLT) (Joint Meeting of the FAO Panel of Experts on Pesticide Residues in Food and the Environment and the WHO Core Assessment Group (2000: Geneva, Switzerland), 2001; Geneva *et al.*, 2001). We implemented and qualified a rat-based PBK model of DLT. Subsequently, these findings were translated to a human PBK model. We further extended the human PBK model to the population of pregnant women and implemented the model in PK-Sim (Dallmann *et al.*, 2018b; Kuepfer *et al.*, 2016; Lippert *et al.*, 2019; Willmann *et al.*, 2003), which is a fundamental step to predict fetal brain exposures. To address uncertainties, we designed case studies with increasing complexity and changes of key model parameters, such as penetration rates of the blood-brain and the blood-placenta barriers (BPBs), which considerably impact DLT uptake and distribution. This work has demonstrated that an integrative approach of quantitative experimental data and computational models can be leveraged to predict human fetal brain levels after DLT exposure and enable an impact assessment of new *in vitro* DNT data on the current risk assessment approach for DLT.

## Materials and methods

###  

#### PBK model development for DLT

**Figure 1.**
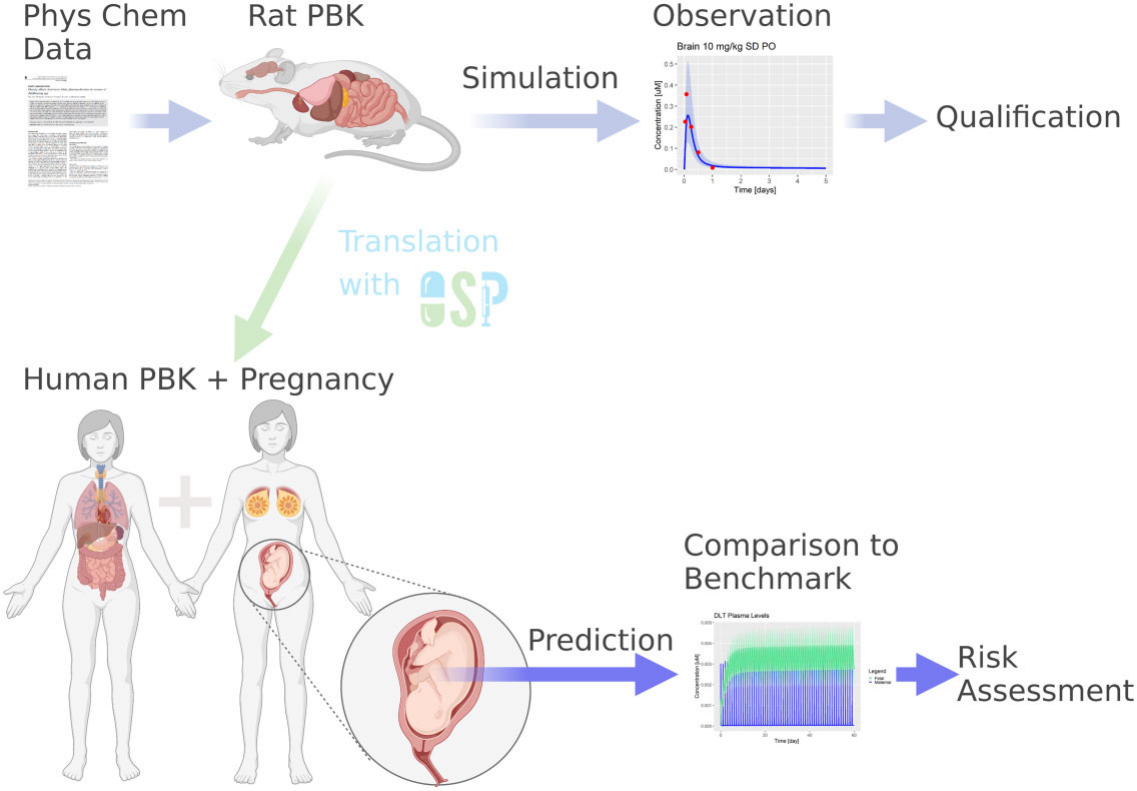
Schematic overview of model development, data curation, and final PK simulations. Firstly, we screen available *in vitro* data and literature available to identify key ADME-related parameters. Secondly, a species-specific PBK model is developed, and potentially refined by comparison to either published models or using reported PK data of the compound of interest. Lastly, the final PBK model can be used to perform a SA as well as simulate PK of compounds in any organ.

We implemented a DLT PBK model in PK-Sim (version 9) (Kuepfer *et al.*, 2016; Lippert *et al.*, 2019; Willmann *et al.*, 2003), an open-source software providing a PBK modeling framework with a highly detailed whole-body structure. Physicochemical properties of DLT curated from the literature and company-internal data (Supplementary Table 1) and available *in vitro* and *in vivo* data in rats (Supplementary Table 2) were used for model development in rats. The generic workflow diagram (Figure 1) depicts the PBK model development process, data curation, and final model simulations to estimate fetal brain exposures.

In PK-Sim, the partition coefficients are calculated using partitioning surrogate measures and any of the 5 different published partitioning methodologies (Berezhkovskiy, 2004; Poulin and Theil, 2002; Rodgers *et al.*, 2005; Rodgers and Rowland, 2006; Schmitt, 2008; Willmann *et al.*, 2003). These methods have been well established for pharmaceuticals, which in general do not exceed lipophilicity values (logKow) of 5. However, some agrochemicals such as DLT have molecular weights greater than 500 g/mol and a logKow value above 5, which may lead to poor absorption and reduced tissue permeation (Krishnan, 2018; Lipinski *et al.*, 2001). In the brain, for example, a lipophilicity beyond logKow of approximately 2–3 may decrease tissue uptake of compounds instead of further improving permeability (Waterhouse, 2003). In addition, increasing lipophilicity typically increases protein binding (eg, to albumin and lipoproteins) of the compound. Due to these binding properties, experimentally derived unbound fraction in plasma (*f*_up_), an important parameter input to PBK models, oftentimes severely overestimate this fraction (Haddad and Poulin, 2015; Watanabe *et al.*, 2018). This results in the overestimation of the total volume of distribution and thus the clearance of the compound. For DLT, the difference of the measured (10% [Sethi *et al.*, 2019]) and the PK-Sim predicted unbound fraction in human plasma (0.0005%, based on logKow = 6.4 [Schmitt, 2008]) is not only significant, but also considerably affects the distribution profile. To adjust for possible mispredictions of the volume of distribution based on the measured *f*_up_, a correction factor for the lipophilicity was introduced and fitted until the model simulations matched available experimental data for rat adipose, brain, fat, liver, and muscle tissues.

##### Rat PBK model development

An adult rat PBK model for DLT was developed and implemented in PK-Sim. The fraction absorbed after oral administration is reported to be at least 0.48 based on *in silico* predictions (Chedik *et al.*, 2017b). In rats, a gastrointestinal absorption of 75% was estimated for DLT by comparing the fraction of radioactivity excreted after a single oral dose versus a single intravenous dose (Bayer Report, 1993). The apparent permeability of DLT in CACO-2-cells shows a moderate bias toward influx (influx ratio: 2.48, internal data; similar values were found in Zastre *et al.* [2013]), with an apparent permeability (*P*_app_) ranged between 4.34E−6 cm/s in the A–B direction and 1.76*E*−6 cm/s in the B–A direction (Supplementary Table 1; A = apical, B = basolateral side).

Influx/efflux transporters are not considered in the PBK model, as none are identified as relevant for DLT to the best of our knowledge. DLT is neither a substrate of ATP binding cassette subfamily B member 1 (ABCB1, or MDR1, P-gp) (Mallick *et al.*, 2020) nor are any other transporters majorly involved in altering influx/efflux such as ATP binding cassette subfamily G member 2 (ABCG2, ie, BCRP) (Amaraneni *et al.*, 2016; Zastre *et al.*, 2013). Moreover, DLT does neither inhibit solute carrier family 22 member 1 (SLC22A1, ie, OCT1) (Bircsak *et al.*, 2013) nor ATP binding cassette subfamily C member 2 (ABCC2, ie, MRP2) (Chedik *et al.*, 2017a).

Metabolism of DLT was informed via expression of cytosolic carboxylesterases (CES) CES1 in microsomal, cytosolic, and plasma spaces, and cytochrome P450 (CYP) 1A2 using the values, as presented in Table 1 (Song *et al.*, 2019). Microsomal and cytosolic CES1 account for the largest fractions metabolized, while the remaining fractions are divided across the main CYP enzymes, mostly CYP1A1, CYP1A2, and CYP2C11 (Anand *et al.*, 2006; Mallick *et al.*, 2020). Both CYP1A2 as well as CES1 expressions are based on the PK-Sim human gene expression database. A correction factor for *K*_m_ and *V*_max_ was implemented to account for discrepancies between the translation from cell-based assays to *in vivo* as described elsewhere (Mallick *et al.*, 2020).

**Table 1.** Overview of metabolism-related parameters implemented in the rat and human PBK model (Song *et al.*, 2019)

Enzyme	Location	*K*_m_ (µM)	*V*_max_ (µM/min)
CES1	Cytosol	Rat: 0.93	Rat: 0.12
		Human: 1.18	Human: 138.13
CES1	Microsome	Rat: 0.76	Rat: 0.10
		Human: 3.81	Human: 364.60
CES1	Plasma	Rat: 1.79	Rat: 0.66
CYP1A2	Mainly liver	Rat: 0.76	Rat: 1.09

*Note*: CYP, cytochrome, *K*_m_, Michaelis–Menten constant; *V*_max_, maximum rate of metabolism.

Experimentally derived tissue PK data in adult rats (experiments used postnatal day [PND] 90 rats) for DLT dissolved in glycerol formal for oral application via gavage were available for plasma, brain, liver, adipose, and muscle tissue after 1, 2, and 10 mg/kg, as well as 0.5 mg/kg after IV dosing (Kim *et al.*, 2008; Mortuza *et al.*, 2018; Song *et al.*, 2019) and used for model parameter estimation. The parameters identified for the fitting process include the partition coefficients and correspondingly the permeability values across the epithelium layer, the lipophilicity correction factor, a metabolism correction factor, and the shape, as well as the time, of dissolution (to match the dissolution profile of DLT after oral absorption in rats). Values for these parameters were estimated simultaneously by minimizing the difference between the observed experimental rat TK data and the model simulations using a least-square approach. In addition, further dose levels and solvents, which were not included in the fitting process (Williams *et al.*, 2019), were tested to qualify the model’s performance to predict the kinetics of DLT in rats.

##### Translation to human PBK model

The developed rat PBK model of DLT was translated to humans with the aim to estimate the human fetal brain exposure (*in vitro*-*in vivo* extrapolation in combination with a species extrapolation). The rat physiology was exchanged for human physiology in PK-Sim to develop a human base PBK model of DLT. We assumed the same key physiological processes implemented in the rat PBK model to be valid in the human model. There is no literature evidence that indicates, for example, the involvement of transporter (eg, in the human BBB) that needs to be taken into account in translating the rat to the human model. However, metabolic parameters (*V*_max_ and *K*_m_ values; Table 1) were adapted, especially considering that CYP enzymes contribute less to the metabolism of DLT in humans than in rats. The majority of metabolism in humans is assigned to CES1 enzymes and only tissue CES expression (microsomal and cytosolic but not in plasma) was accounted for as it covers 97% of metabolism in humans (Anand *et al.*, 2006; Hedges *et al.*, 2019). A scaled apparent intrinsic total clearance value of 646 ml/min/g liver was reported for metabolism of DLT in adult human liver (Hedges *et al.*, 2019).

##### PBK maternal-fetal model

In addition to the base PBK model, a maternal-fetal PBK model extension was implemented to simulate the systemic concentrations of exposure to DLT in the fetus. The maternal-fetal PBK model extension is publicly available and open-source in a series of recent publications (Dallmann *et al.*, 2017a,b, 2018a,b; Mian *et al.*, 2021).

For a detailed description of the workflow on the extension of a base PBK model for pregnant women within PK-Sim and MoBi, we refer the reader to the tutorial by Dallmann *et al.* (2018b).

##### Estimation of fetal brain exposure

**Figure 2.**
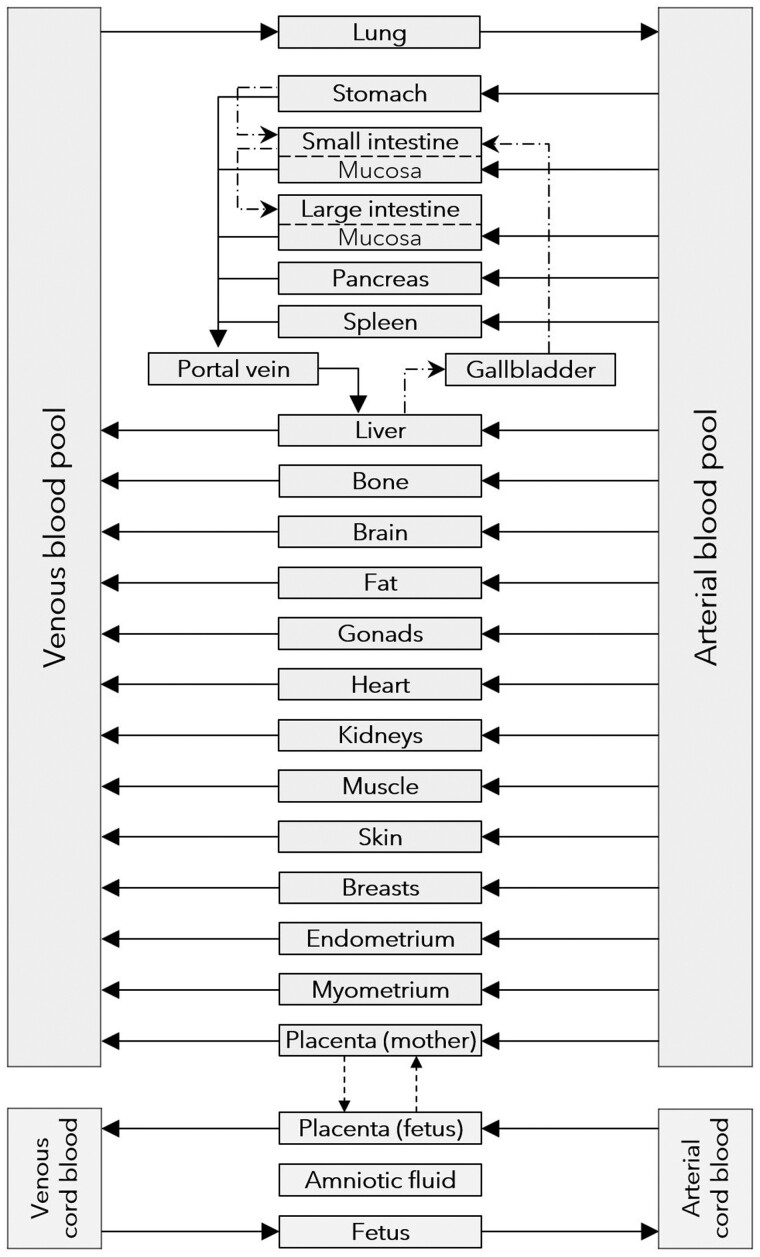
Structure of the human PBK maternal-fetal model (from https://github.com/Open-Systems-Pharmacology/Pregnancy-Models/blob/master/ModelStructure.png). Solid arrows represent blood flow, while dashed arrows indicate passive diffusion of compounds.

The implemented maternal-fetal PBK model includes all pregnancy-relevant compartments, as well as fetal plasma and lumped tissue compartments, that is, the model provides no further distinction between developing organs in a fetus (Figure 2). Therefore, the fetal brain exposure is approximated by using the following equation:


(1) $${C\left(\mathrm{brain}\right)}_{\mathrm{fet},\max}=\frac{{{C\left(\mathrm{plasma}\right)}_{\mathrm{fet},\max}*C\left(\mathrm{brain}\right)}_{\mathrm{mat},\max}}{{C\left(\mathrm{plasma}\right)}_{\mathrm{mat},\max\;}},\;$$

where “mat” and “fet” refer to maternal and fetal compartments, respectively, *C*() is the simulated concentration in brain and plasma, and “max” indicates the maximum observed concentration. This equation assumes the same compound ratio between brain and plasma in the mother and the fetus. Thus, this represents a relative translation of the brain exposure of DLT in the mother to the fetus. Alternatively, the developed maternal-fetal PBK model provides the possibility to simulate the exposure of DLT in fetal tissue (Figure 2). While this compartment represents the fetus’s total tissue, it may serve as an approximation for brain concentrations. In general, the simulated tissue concentrations are below the calculated fetal brain concentrations (equation 1). For a more conservative estimate of the fetal brain exposure and therefore the neurotoxic risk, both calculation and simulation approaches were used to inform a range of possible fetal brain exposures.

The determination of *C*(plasma)_fet, max_ and *C*(brain)_mat, max_ including the strategy to address uncertainties is described in the following paragraphs.

#### Model evaluation

##### DLT transfer rates across the blood-brain barrier

There are limited data on DLT’s pharmacokinetics in humans to estimate concentration ratios between plasma and brain tissue levels, which could inform DLT distributions in the model. Therefore, a value was derived, based on *in vivo* rat data or alternatively by using quantitative structure-activity relationship (QSAR) models based on physicochemical descriptors. We used QSAR to estimate the potential range of ratios between plasma and brain tissue levels as a proxy for BBB integrity and with that to address the uncertainty of the concentration ratio in humans. Based on available QSAR models (Bujak *et al.*, 2015), 5 models were selected for use to estimate the brain-plasma concentration ratio (Table 2). A range of 0.37–1.5 was obtained for the brain-plasma concentration ratio by the 5 QSAR models based on DLT physiochemical properties. Further, a recent study using *in vivo* rat data estimated this value at 0.44 (Mallick *et al.*, 2020). A value of “1” was used as proxy for an impaired BBB and “1.5” was used as a hypothetical worst-case scenario (according to QSAR models).

**Table 2.** Estimated human brain–plasma concentration ratios of DLT

Reference	Descriptor	Ratio	Comment
(Kaliszan, 1996)	log*P*, MW	0.37	Calculated
(Takaku *et al.*, 2015)	MW, PSA, *H*_max_	0.4–0.6	(@ *H*_max_ range) Calculated
(Clark, 1999)	PSA	0.7	Calculated
(van de Waterbeemd and Gifford, 2003)	PSA, molecular volume	0.58	Calculated
(Pan *et al.*, 2004)	log*P*, PSA	1.5	Calculated
(Mallick *et al.*, 2020)	–	0.44	Fit

*Note*: logKow, lipophilicity; MW, molecular weight; PSA, polar surface area; *H*_max_, maximum energy-state of hydrogen atom.

In our approach, the rat PBK model was fitted to available experimental data to obtain a proxy for human maternal brain plasma (*C*_max_) ratio. A value of 0.06 was obtained, which was then also taken as the value for the fetal brain (see Table 3) and considered as a realistic scenario.

**Table 3.** Overview of model application scenarios (Cases 1–7)

Case	Maternal BP PC	Achieved maternal	Fetal/maternal PR	Solubility	Fetal age (weeks)
		**BPR (*C*_max_)**	(Dallmann *et al.*)	**(mg/l)**	
DD + low sol.	0.17^a^	0.06^b^	0.2^c^	2.7*E*−3^d^	20
DD				323^a^	
DD + no BBB	65	1^e^	0.2^c^		
DD + no BPB	0.17^a^	0.06^b^	1^e^		
DD + no BBB + no BPB	65	1^e^	1^e^		
Worst	100				
Worst + 40 weeks		1.5^f^	1.2^c^		40

*Note*: BBB, blood-brain barrier; PC, partition coefficient; *C*_max_, concentration maximum; DD, data-driven; Mat., maternal; fet., fetal.

a Fitted.

b Fitted PC results to achieve that ratio.

c Median (read-across).

d In-house data from Bayer; experiment in water.

e Fully impaired.

f QSAR model (cf. “Materials and methods” section).

##### DLT transfer rates across the BPB

A read-across approach (Escher *et al.*, 2019) was used to define a range of blood-placenta partitioning for DLT. Six compounds were selected from a comprehensive list on human *in vivo* fetal/maternal plasma concentration ratios collected from public literature (Supplementary Table 4). The 6 compounds reflect the properties of DLT such as the molecular weight >400 g/mol and lipophilicity (logKow) >3.5 (Table 4). Based on this comparison, a median fetal/maternal plasma ratio of 0.2 (range, 0–0.35) was obtained. For a hypothetical worst-case scenario, a median fetal/maternal plasma ratio of 1.2 (with a range of 1–5) was calculated from this list from all compounds, with a fetal/maternal plasma ratio >1 (higher concentration in the fetal than the maternal plasma).

**Table 4.** Overview of compounds that provide comparable physicochemical properties to DLT for the estimation of fetal/maternal concentration ratios

Compound	MW (g/mol)	logKow	Fetal/maternal PR	References
Atazanavir	705	4.08	0.14	(Boffito *et al.*, 2004; Ripamonti *et al.*, 2007)
Buprenorphine	468	3.93	0.14–0.35	(Concheiro *et al.*, 2010a,b; Gordon *et al.*, 2010; Jensen *et al.*, 2007)
Lopinavir	629	3.91	0.22	(Lambert *et al.*, 2011; van Hoog *et al.*, 2012)
Nicardipine	480	3.82	0.01	(Bartels *et al.*, 2007; Benet *et al.*, 2003)
Nelfinavir	568	4.61	0.02	(Boffito *et al.*, 2004; Gingelmaier *et al.*, 2006; van Hoog *et al.*, 2012)
Ritonavir	721	4.30	0.45	(Gingelmaier *et al.*, 2006; Law *et al.*, 2004)

#### PBK model simulations

The model was used to estimate human fetal brain exposures across various case studies, with variations of key model assumptions and parameters (eg, blood-brain and BPB partitioning) assessed to address uncertainties. For all scenario-based evaluations, the human PBK model was run until a steady state was reached (approximately 60 days). The ADI of 0.01 mg/kg/day was used as the reference dosing scenario. A pregnant population of 100 women was simulated, which is considered sufficient to adequately capture population variability in physiology and anatomy. An overview of the performed model scenarios can be found in Table 3.

The maternal brain-plasma partition coefficient describes “how much” compound can theoretically be retained in the brain relative to plasma fluids. In contrast, the maternal brain-plasma concentration ratio (*C*_max_) reflects the ratio of effectively achieved *C*_max_ in brain tissue and plasma during the (simulated) exposure scenario. This ratio is smaller than the partition coefficient, as it depends on “how fast” (permeability) the compound can cross the barrier. If concentrations rise and fall quickly, but permeability is low, the *C*_max_ ratio tissue/plasma will be smaller than the tissue/plasma partitioning, as kinetics in the brain are “delayed” by permeation.

For cases 1, 2, and 4, the maternal brain/plasma ratio of 0.06 is a result of species extrapolation from rats using the brain partition coefficient (0.17) fitted with rat TK data, which is lower than the values estimated by the QSAR models. In Table 3, the column, ‘fetal/maternal plasma ratio’, reports plasma *C*_max_ ratio values in maternal and fetal plasma according to the read-across approach described above and is also dependent on both partitioning and permeability.

The permeability across the BPB was estimated, such that the steady-state concentrations of DLT in fetal and maternal plasma match the reported ratio (Table 3). Likewise, the maternal brain partition coefficient for cases 3, 5, 6, and 7 was estimated, such that the simulated maternal brain/plasma ratio (Table 3) would equal to 1 or 1.5, indicating impaired functionality of the BBB. Cases 6 and 7 only differ in the gestational age, which is an important factor as the currently implemented version of the maternal-fetal PBK model does not allow to model a growing fetus. When simulating the PBK model for, for example, 60 days, the fetus stays at the same age over the simulation period. Thus, we chose to investigate further the effect of a more developed over a lesser developed fetus to estimate the fetal brain concentrations and chose 20 and 40 weeks. The *in vitro* BMCs were obtained in an *in vitro* model based on human neural progenitor cells generated from primary human brain at gestational weeks 16–19 (Masjosthusmann *et al.*, 2020) and matured for 3–4 weeks. Thus, this corresponds to the selected value of 20 weeks for the gestational age in the PBK model.

Additionally, we performed a sensitivity analysis (SA) to identify model parameters affecting the maternal brain and fetal plasma concentrations.

## Results

###  

####  

##### Rat PBK model simulations

**Figure 3.**
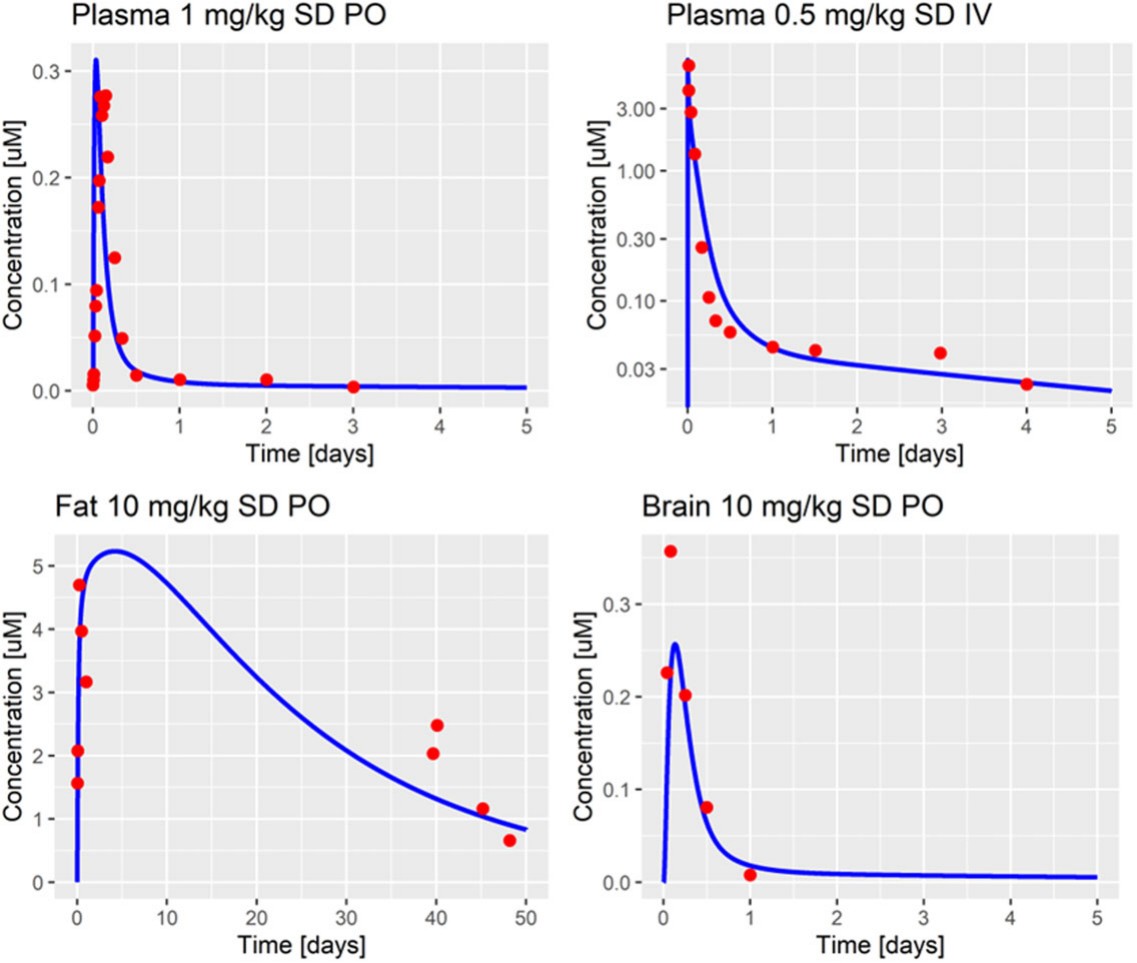
Model simulation (solid blue line) and adult rat (PND90) PK data (red dots) in plasma after a single oral dose of 1 mg/kg (top left), single IV dose of 0.5 mg/kg (top right; data taken from Mortuza *et al.* [2018]), after a single dose of 10 mg/kg in fat tissue (bottom left), and brain tissue (bottom right; data taken from Song *et al.* [2019]). SD, single dose.

The model describes the observed plasma concentrations in adult (PND90) rats well for a single oral dose of 1, 2, and 10 mg/kg. When simulating an IV infusion of 0.5 mg/kg, the model further captures the observed data well. The resulting model fits are shown in Figure 3. Even long-term PK data for fat tissue can be captured. On the other hand, the model slightly underpredicts the observed maximum concentration in the rat brain after a single dose of 10 mg/kg. The simulations for liver, muscle, and fat at 2 and 10 mg/kg are shown in the Supplementary Material (Supplementary Figure 10). In general, the over- and underprediction of the observed maximum concentrations in plasma and tissues are within a 2-fold range of the simulated values and are thus considered to be acceptable at 1, 2 and 10 mg/kg.

In summary, the model fitting process resulted in parameter values (Table 5), which allowed the description of observed PK data in adult PND90 rats over a wide range of doses (0.5–10 mg/kg) and from a variety of published studies (Kim *et al.*, 2008; Mortuza *et al.*, 2018; Song *et al.*, 2019). Parameters were also optimized to describe the experimental setup, including the dissolution of DLT in glycerol formal as a carrier. Further, the model simulations are in line with a recently published rat minimal PBK model (visual comparison [Song *et al.*, 2019]).

**Table 5.** Parameter values (optimal) after fitting the rat PBK model to experimental PK data

Parameter	Unit	Optimal value	Min value	Max value
Lipophilicity	a.u.	1.8	1.5	6.4
Correction factor for lipophilicity a.u.		3.6	1	4.3
Solubility	mg/l	323.2	0.01	1000
Metabolism correction factor	a.u.	4.3	1	10
PC brain	a.u.	0.2	0.01	10
PC fat	a.u.	172.8	1	1000
PC liver	a.u.	8.00	0.1	100
PC muscle	a.u.	4.25	0.1	100
Perm brain	cm/min	10	1*E*−7	10
Perm fat	cm/min	2.5*E*−5	1*E*−7	10
Perm liver	cm/min	2.8*E*−6	1*E*−7	10
Perm muscle	cm/min	2.5*E*−5	1*E*−7	10
Dissolution shape (Weibull)	a.u.	0.01	1*E*−3	5
Dissolution time (Weibull)	min	1000	1*E*−3	1000

*Notes*: The columns minimal and maximum value provide lower and upper boundaries for the algorithm. PC, partition coefficient; Perm, permeability; a.u., arbitrary unit.

##### Rat PBK model validation

We then tested the model’s performance to predict the kinetics of DLT at other dose levels and solvents, which were not included in the fitting process (Williams *et al.*, 2019). The model predictions were compared with observed PK data in plasma and brain after 8 and 25 mg/kg single administrations of DLT dissolved in corn oil in adult rats. As shown in Supplementary Figure 11, the model overpredicts the observed PK. This is likely due to the difference in formulation with corn oil (and the larger volume of 5 ml/kg administered) and glycerol formal. Previous studies have shown that DLT administered in larger volumes of corn oil resulted in a retard absorption within the gastro intestinal tract and therefore in a lower *C*_max_ (Mortuza *et al.*, 2018). Thus, the model setup, calibrated for glycerol formal, provides an upper limit (ie, worst-case evaluation) for estimating a maximum simulated concentration for safety assessments.

##### Human PBK model simulation

The developed human PBK model was simulated to estimate the PK of DLT in humans, focusing on maternal-fetal kinetics, for a number of scenario-based evaluations as described (Table 3).

##### Effect of DLT solubility

**Figure 4.**
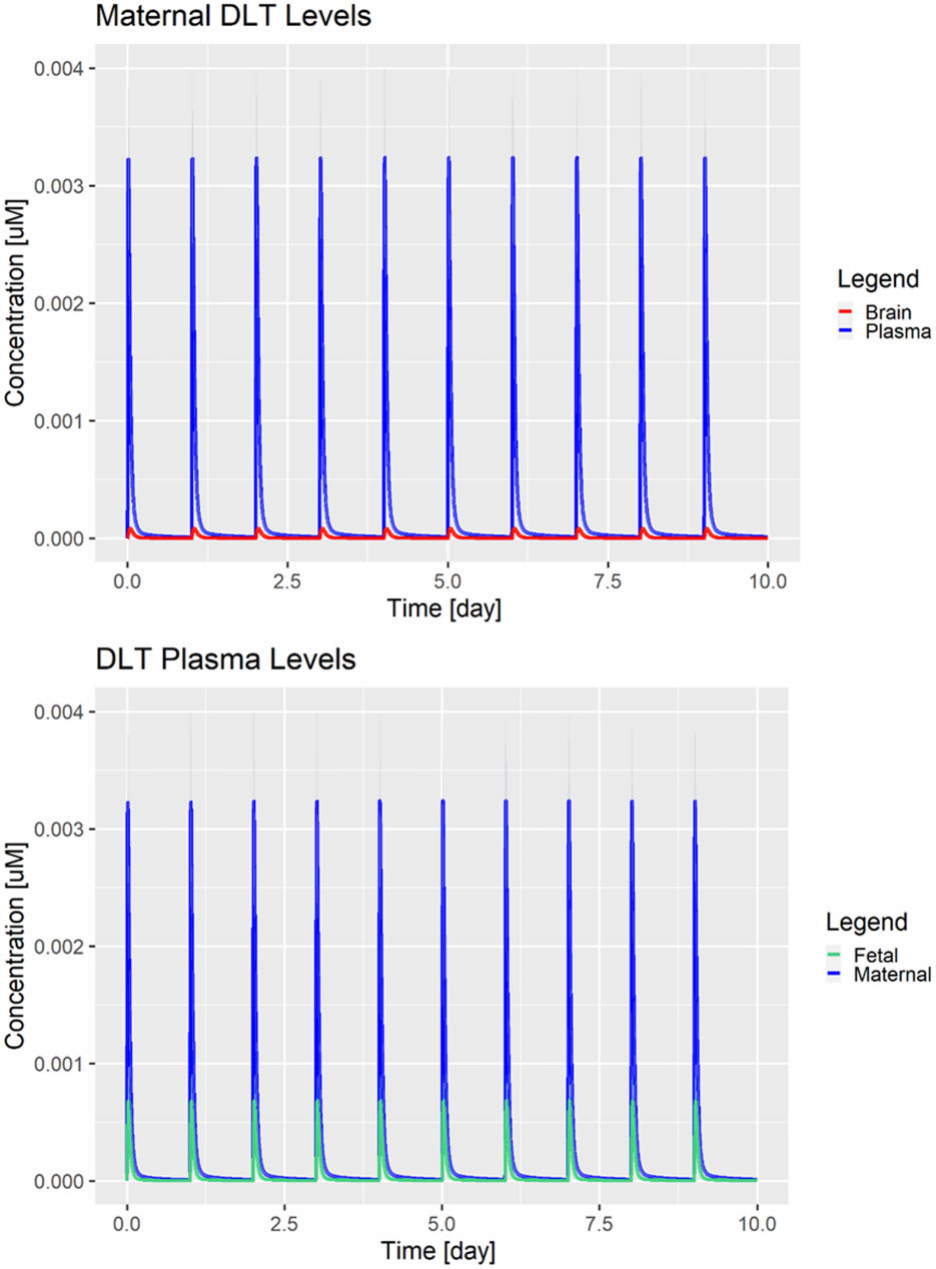
Model simulations for the data-driven scenario (Case 2) after repeated single daily administrations of 0.01 mg/kg (ADI) for maternal brain (red), maternal plasma (blue), and fetal plasma (green). Solid lines represent the population means over 100 simulations and shaded areas represent 1 standard deviation.

The effect of increasing the solubility of DLT from the value dissolved in water (Supplementary Table 1; < 1*E*−3 mg/l) to the fitted value of DLT (Table 5; 323 mg/l) formulated with glycerol-formal is shown in Supplementary Figure 4 and Figure 4 (Cases 1 and 2). Overall, the simulated concentration maximum (compare *y*-axis) in both maternal and fetal plasma and brain compartments is comparable. Potentially, at such low administered doses (ADI = 0.01 mg/kg/day), solubility is not the rate-limiting step in the absorption. The compound is readily dissolved, absorbed, and then distributed in the systemic circulation and subject to tissue uptake and metabolism. In contrast, at higher doses, the effect of solubility would become more apparent as absorption of a nondissolved compound can be limiting. Simulated and estimated fetal brain concentrations are 28.000–95.000 times below the *in vitro* BMC of 0.5 µM (Table 6). Note that the maternal plasma/brain concentration ratio is about 0.06, indicating limited transfer of DLT across the BBB, which is based on the *in vivo* rat data. Likely, this is due to the tight junction formation in the BBB as well as the absence of any known active uptake transport process across the BBB (Pardridge, 2012).

**Table 6.** Estimated fetal brain exposures for all model applications after ADI dosing and MoS relative to the benchmark of 0.5 µM

Case	Calc. *C*_max_ FB (µM)	Sim. *C*_max_ FT (µM)	MoS (Thr./brain)	MoS (Thr./tissue)
DD + low sol.	15*E*−6	5*E*−6	34 349	95 639
DD	18*E*−6	6*E*−6	28 250	78 311
DD + no BBB	676*E*−6	244*E*−6	739	2051
DD + no BPB	80*E*−6	29*E*−6	6270	17 089
DD + no BBB + no BPB	3137*E*−6	1144*E*−6	159	437
Worst	5664*E*−6	2053*E*−6	88	244
Worst + 40 weeks	6260*E*−6	3481	80	144

*Note*: DD, data-driven; sol., solubility; Calc., calculated; sim., simulated; Thr., *in vitro* threshold (0.5 µM); FB, fetal brain; FT, fetal tissue; BBB, blood–brain barrier; *C*_max_, concentration maximum.

Further, Cases 1 and 2 (Supplementary Figure 4 and Figure 4) represent the direct translation of the developed rat PBK model to humans. However, the implemented solubility of DLT in water seems unlikely to adequately represent the *in vivo* situation (Case 1; see Supplementary Material for elaborations on solubility). Case 2 is considered to represent the solubility behavior within the human gastro-intestinal tract more appropriately as solubility of DLT was explicitly estimated.

##### Impaired blood-brain and BPB

We further investigated the effect of impaired (ie, higher transfer of DLT into brain or placenta) maternal blood-brain (case 3) or BPBs (case 4) and a combination of both (case 5) to cover variability in permeability parameter values for both barriers. Further, the SA identified parameters related to the barriers to be among the most influential parameters to affect fetal DLT concentrations (Supplementary Material).

The results are shown in Supplementary Figures 5–7. In case of any impairment of the respective barriers, plasma (maternal/fetal) and plasma/brain concentration ratios are comparable and reflect a ratio of 1. The margin of safety (MoS) ranges between 160 and 17 000 (Table 6).

##### Worst-case scenarios

**Figure 5.**
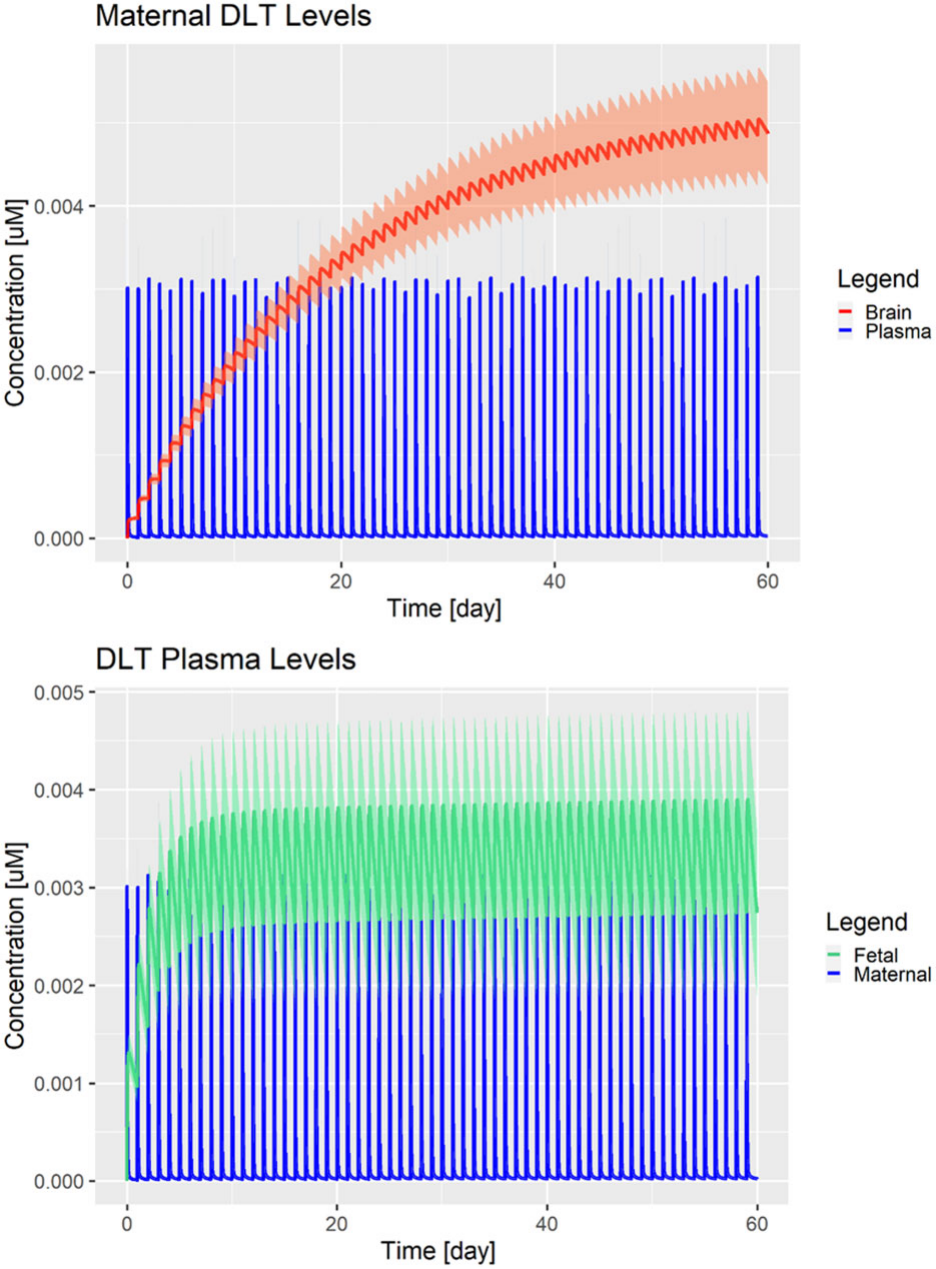
Model simulations for the worst-case scenario (Case 7) after repeated daily administrations of 0.01 mg/kg (ADI) for maternal brain (red), maternal plasma (blue), and fetal plasma (green). Solid lines represent the population means over 100 simulations and shaded areas represent 1 standard deviation.

The worst-case scenarios (Cases 6 and 7) consider brain-plasma and fetal/maternal plasma ratios above 1 as input parameters, and, for the last scenario, even a fetus at gestational age of 40 weeks. Both scenarios show DLT accumulation in the brain, while the last scenario (Case 7) shows also a slight accumulation of DLT in the fetal plasma (Figure 5) at gestational age of 40 weeks. This accumulation effect is not observed with a younger fetus with an age of 20 weeks (Supplementary Figure 8; Case 6). However, human fetal brain concentration is still far below the BMC.

For graphical purposes only, a finer resolution of the last 10 simulation days for cases 3, 5, and 6 is provided in the Supplementary Material (Supplementary Figures 5, 7, and 8).

In a hypothetical worst-case scenario, which includes no BBB and no BPB, with a gestational age of 20 and 40 weeks, the MoS closes in on the threshold if an exposure of 0.9 mg/kg/day is considered, which is at least 90 times higher than the acceptable exposure to residues of DLT as active substance in plant protection products.

##### Quantitative evaluation of fetal brain exposure

An overview of the estimated human fetal brain exposures (equation 1) and the simulated human fetal tissue levels of DLT in steady state after multiple daily administrations of DLT at the ADI up to 60 days is presented in Table 6.

The MoS relates the reported *in vitro* threshold of 0.5 µM to the calculated and simulated human fetal brain concentrations of DLT. Here, the data-driven approach (Case 2) results in a high MoS of 78 311. When dosing at the ADI levels of 0.01 mg/kg/day, the MoS, even in the worst-case scenario, is still between 80 and 244, indicating a simulated fetal brain DLT concentration orders of magnitude lower than the *in vitro* benchmark.

The SA confirmed that lipophilicity, BPB permeability, and brain partitioning are among the most impactful parameters to change concentration maximum in brain and fetus, justifying the relevance of the chosen case scenarios (Supplementary Figures 1 and 2).

## Discussion

The aim of the present study was to compare the human fetal brain concentration under realistic worst-case *in vivo* exposure conditions (0.01 mg/kg bw/day, ADI for DLT) with the lowest BMC (approximately 0.5 µM [Masjosthusmann *et al.*, 2020]) achieved in the *in vitro* DNT testing battery by exploiting a PBK model.

We demonstrated that total DLT concentrations in the fetus will likely be far below (MoS = 78 311 [realistic scenario] − 80 [worst-case scenario]; Table 6) the *in vitro* benchmark level of the developmental neurotoxic test battery (Masjosthusmann *et al.*, 2020). When considering unbound, that is, free DLT concentrations, these will likely be even lower and, therefore further below the benchmark level.

To this end, we first developed a rat PBK model to describe the pharmacokinetics of DLT *in vivo*. We identified and estimated key model parameters (lipophilicity, solubility, and partition coefficients) that are essential to recapitulate DLT kinetics over a variety of dose ranges and published studies.

Previous reports demonstrate the ability to recapitulate PK data of DLT in rats and showed promise to simulate kinetics in humans (Mallick *et al.*, 2020; Song *et al.*, 2019), whereas in this investigation we deliberately included more PK data, more mechanistic detail and biological processes, and designed different scenarios to capture uncertainties in key model parameters.

We then translated the PBK model to the special population of pregnant women, with the aim to predict DLT kinetics in the human fetus. The primary focus is to aid in risk assessment of fetal exposures to DLT. Due to the lack of data on DLT levels in humans and to address uncertainties in estimating DLT levels in the fetus, we have designed case studies (Cases 1–7) to capture the effect of, for example, DLT solubility, an impaired blood-brain or BPB either individually or in combination. We further extended this approach by altering the fetal age and a potential accumulation of DLT in the brain over time (worst-case scenario). Each case study was chosen to represent a different driver thought to be most relevant in DLT uptake. This was confirmed by a SA (Supplementary Figures 1 and 2), which identified lipophilicity, BPB permeability, and brain partitioning as the most impactful parameters to alter maximum concentrations in the maternal brain and fetal plasma. The gastro-intestinal uptake (solubility) of DLT strongly depends on the administered vehicle and is high in glycerol formal (Mortuza *et al.*, 2018), for which the rat PBK model was parameterized. Consequently, we tested and confirmed this by over-predicting reported PK data on DLT dissolved in corn oil (exhibiting a lower gastro-intestinal absorption; Supplementary Figure 11).

In a data-driven and thus more realistic simulation scenario of the developed maternal-fetal PBK human model (Case 2), the MoS between the *in vitro* BMC and the predicted *in vivo* concentration spans over 5 orders of magnitude. Further, Cases 3–5 presented a scenario to simulate the impairment of either BBB, BPB, or the combination. In any scenario, the MoS ranged between 160 and 17 000.

Although considered unrealistic based on the available experimental data, to drive an even more extreme scenario, we further explored the possibility of DLT accumulation in the fetus and the effect of an aging fetus (gestational weeks 20–40) on DLT exposures in the fetal brain. This resulted in a pronounced drop of the MoS. Strikingly, the estimated brain concentration is still at least 80 times below the benchmark level.

In a recent investigation by Personne *et al.* (2021), rats were exposed to another pyrethroid, cis-permethrin, during gestation and fetal and maternal blood and brain levels of cis-permethrin were analyzed. The toxicokinetic profiles showed a lower exposure to cis-permethrin in fetal blood and fetal brain compared with dams which is further strengthening the selection of Case 2 as a realistic scenario.

To put the MoS in perspective, it needs to be highlighted here that a value of 100 is normally used to derive an ADI if it is derived from the no observed adverse effect level from an animal study (International Programme on Chemical Safety and IPCS Workshop on Incorporating Uncertainty and Variability into Risk Assessment (2000: Berlin, Germany), 2005; Berlin, 2005). The value of 100 consists of a factor of 10 for both, interspecies and human interindividual differences, which is further divided into toxicodynamic and toxicokinetic differences. Therefore, by using human *in vitro* data and a human PBK model for risk assessment, the default safety factor of 100 would be overconservative and scientifically not justified as no species extrapolation is required as well as biological variability in toxicokinetics is already partly covered within the model.

Despite being able to recapitulate DLT PK data in rats and to demonstrate that the developed PBK model can be used to efficiently predict potential fetal exposures to DLT, we acknowledge that our model has limitations and do recognize the biological complexity. The PBK model in PK-Sim is built around the concept of albumin or alpha-glycoprotein binding to estimate the fraction unbound of DLT in plasma. However, currently, it does not explicitly model effects of lipid or lipoprotein binding-related factors on compound distribution, which, for DLT, accounts for 20–30% of the bound fraction (Sethi *et al.*, 2019). In this study, we have captured the case of comparable levels of lipoproteins between mother and fetus. Information on the levels of lipoproteins could be well implemented in future versions of the model and help to further assess DLT levels more appropriately.

Additionally, the current approaches implemented in PK-Sim to calculate partition coefficients are based on and tested with pharmaceuticals. They have not yet been widely tested for chemicals, such as pesticides, and a range of high lipophilicity values with a logKow above 5. The direct translation from rat to humans shows a low partitioning of DLT into the human fetal brain with a high MoS (Table 6). Here, the key model assumption is that the PK in rats and humans is comparable. To account for species-dependent differences in DLT tissue uptake, we explicitly designed the simulation scenarios. A distinguishing aspect of this investigation is the powerful application of *in silico* approaches (used to estimate the potential range of ratios between plasma and brain tissue levels) over conventional, experimental studies in generating potential scenarios, especially when clinical data are sparse, making *in silico* approaches a powerful tool in risk assessment.

A key difference to published models is that the here developed model does not predict an accumulation in the human brain in the data-driven scenario (Case 2), and the concentration maximum is lower than simulated by Mallick *et al.* (2020). After doses of 1 mg/kg/day, their model simulation shows accumulation in the maternal brain at around 25 ng/ml (at steady state; which corresponds to about 50 nM DLT). While we simulate at a lower dose (ADI = 0.01 mg/kg/day), we do not observe an accumulation effect in the data-driven scenario. In our model, accumulation in the maternal brain only occurs when the BBB is fully impaired (Case 3), which is an unlikely scenario. These differences in the model results might be due to a different model setup (full vs minimal PBK), the more in-depth representation of physiology (intracellular, interstitial spaces in tissues), as well as the estimated brain permeability in PK-Sim. Also, Mallick *et al.* (2020) do not include a parameterization of partition coefficients via lipophilicity (logKow) or solubility, which drive the absorption of orally dosed DLT. Complementary to these aspects is the observation that the measured brain PK after 2 and 10 mg/kg in rats (Supplementary Figure 10) quickly declines and shows negligible concentrations of DLT 24 h postadministration. It would be anticipated that any additional dosing afterward would likely result in the same PK as observed before and thus not lead to accumulation. However, the lack of clinical data and the potential storage of DLT in adipose tissue hinder a full assessment of the *in vivo* situation in humans. Consequently, modeling DLT TK within a PBK modeling approach remains challenging.

In future, further model optimization could be done to extent the applicability domain in PK-Sim to highly lipophilic compounds. The partition coefficient calculation methods could be expanded, for example, with prior work on the effect of highly lipophilic compounds on the estimation of fraction unbound, the volume of distribution, and the calculation of partition coefficients (Poulin and Haddad, 2012).

In summary, we have demonstrated that the here developed PBK modeling framework is able to recapitulate DLT kinetics in rats, and, when translated to humans can be employed to predict fetal brain exposures.

Our results indicate that the current ADI for DLT is still protective even considering the new *in vitro* DNT IVB results. This approach demonstrates the applicability of the developed PBK model and the possibility to generate fit-for-purpose simulation scenarios which make PBK models a powerful tool in NGRA.

## Supplementary Material

kfad007_Supplementary_Data
