# Supplementary material for: Considering developmental neurotoxicity in vitro data for human health risk assessment using physiologically-based kinetic modeling: deltamethrin case study
Source: Toxicol Sci. 2023 Jan 13;192(1):59–70. doi: 10.1093/toxsci/kfad007 (PMC10025876; doi:10.1093/toxsci/kfad007)
Supplement: kfad007_Supplementary_Data [file kfad007_supplementary_data.docx]

Supplemental Material:

Consideration of developmental neurotoxicity (DNT) *in vitro* data for human risk assessment using physiologically-based kinetic (PBK) modeling: deltamethrin case study

# Solubility

Deltamethrin is lipophilic and poorly soluble in water (1E-3 mg/L). However, water is not the best surrogate solvent for intestinal fluid and not suitable to predict the solubility of poorly soluble drugs in the gut. Innumerous intrinsic and extrinsic factors can play a role in drug solubility, e.g., pH, fed/fasted state, biliary salts, amphiphilic compounds, gastrointestinal fluids volume, viscosity, etc. Therefore, the experimentally derived water solubility value is likely not representative for *in vivo* gastric solubility and could not be used directly as input parameter for the physiological PBK model. The value was corrected (i.e., fitted, see description of correction factor for logKow) such that the experimental TK data in rats (the kinetics during the absorption phase) could be correctly described by the model simulations. The resulting solubility value thus represents the solubility of the carrier-deltamethrin complex, which was used in the animal experiments (glycerol formal). Solubility trials (conducted after the PBK modelling project) with fluids simulating gastro-intestinal fluids confirmed a higher solubility of DLT in intestinal fluids (up to 12 mg/L) compared to water (internal unpublished data). However, this is still within the range of the estimated value of 323 mg/L. Regardless, the experimentally derived values could be used in future computational investigations to inform the modeling process more adequately.

# Data

The following tables provide an overview of the physicochemical properties of deltamethrin (Table S1) as well as published studies, which report PK of deltamethrin in rats (Table S2).

Table S1: Compound properties used as input parameters for the deltamethrin PBPK model.

| PARAMETER | Value | Comment (Reference) |
| --- | --- | --- |
| MW (g/mol) | 505.2 | Molecular weight, 2x Br halogens |
| pKa 1 | N/A | Neutral compound |
| LogK_o:w_ | 6.4 | Octanol/water partition coefficient; Rapporteur Assessment Report 2017 |
| LogK_o:w_ | 4.5 | EFSA (EFSA Panel on Contaminants in the Food Chain (CONTAM) *et al.*, 2018), WHO (IARC publication deltamethrin 1996) |
| Solubility (mg/L) | > 1E-3 (in water) | Rapporteur Assessment Report 2017, no reports identified for biorelevant media at conduct of PBK modelling |
| BPrat | 1.1 | Blood-plasma-ratio; (Mallick et al. 2020) |
| fu_human_ (%) | 10 | Fraction unbound (Sethi *et al.*, 2019) |
| fu_rat_ (%) | 20 | Fraction unbound (Sethi *et al.*, 2019) |
| PSA (Å) | 59.3 | (Topological) polar surface area; <https://pubchem.ncbi.nlm.nih.gov/compound/Deltamethrin> |
| H_max_ | 0.2-1.4 | Maximum energy state of hydrogen atom, taken from (Takaku *et al.*, 2015) |
| P_app_ (cm/s) | AB: 4.34E-6  BA: 1.76E-6 | Apparent permeability across a CACO-2-monolayer; A = apical, B = basolateral side (Zastre *et al.*, 2013) |

Table S2: Overview of literature reports contributing data to the development of the PBPK model for rats and humans

| ID | Study | Description | Substance | Species | Comments |
| --- | --- | --- | --- | --- | --- |
| 1 | Kim et al (2010) | Plasma and tissue PK @ 0.4, 2, and 10 mg/kg (all PO) single dose | Deltamethrin | Rats | DLT dissolved in glycerol formal |
| 2 | Mortuza et al (2018) | Plasma PK @ 0.5 mg/kg (IV) and 1 mg/kg (PO) single dose | Deltamethrin | Rats | DLT dissolved in glycerol formal |
| 3 | Hedges et al (2019) | Metabolism of DLT in rat and human liver microsomes | Deltamethrin | Rats, Humans |  |
| 4 | Song et al (2019) | Pyrethroid Rat PBPK model development | Deltamethrin, cis- and trans-permethrin | Rats | IVIVE approach |
| 5 | Williams et al (2019) | Plasma and brain PK after 8 and 25 mg/kg single dose PO | Deltamethrin | Rats | DLT dissolved in corn oil |

# Final Model Parameters

Table S3: Overview of final model parameter values for each model scenario

| Parameter | Unit | Case 1 | Case 2 | Case 3 | Case 4 | Case 5 | Case 6 | Case 7 | Comment |
| --- | --- | --- | --- | --- | --- | --- | --- | --- | --- |
| Lipophilicity Correction Factor | a.u. | 3.6 | 3.6 | 3.6 | 3.6 | 3.6 | 3.6 | 3.6 | Fitted |
| Solubility | mg/l | 2.70E-03 | 323.19 | 323.19 | 323.19 | 323.19 | 323.19 | 323.19 | Fitted |
| CES reference concentration | uM | 0.23 | 0.23 | 0.23 | 0.23 | 0.23 | 0.23 | 0.23 | Fitted |
| PC Brain (Maternal) | a.u. | 0.17 | 0.17 | 65 | 0.17 | 65 | 100 | 100 | Fitted |
| PC Fat | a.u. | 172.78 | 172.78 | 172.78 | 172.78 | 172.78 | 172.78 | 172.78 | Fitted |
| PC Liver | a.u. | 8 | 8 | 8 | 8 | 8 | 8 | 8 | Fitted |
| PC Muscle | a.u. | 4.25 | 4.25 | 4.25 | 4.25 | 4.25 | 4.25 | 4.25 | Fitted |
| PC Placenta | a.u. | 0.03 | 0.03 | 0.03 | 0.2 | 0.2 | 0.25 | 2.35 | Fitted |
| Perm Brain | cm/min | 10 | 10 | 10 | 10 | 10 | 10 | 10 | Fitted |
| Perm Fat | cm/min | 2.50E-05 | 2.50E-05 | 2.50E-05 | 2.50E-05 | 2.50E-05 | 2.50E-05 | 2.50E-05 | Fitted |
| Perm Liver | cm/min | 2.80E-06 | 2.80E-06 | 2.80E-06 | 2.80E-06 | 2.80E-06 | 2.80E-06 | 2.80E-06 | Fitted |
| Perm Muscle | cm/min | 2.50E-05 | 2.50E-05 | 2.50E-05 | 2.50E-05 | 2.50E-05 | 2.50E-05 | 2.50E-05 | Fitted |
| Perm Placenta | cm/min | 1.00E-02 | 1.00E-02 | 1.00E-02 | 1.00E-02 | 1.00E-02 | 1.00E-02 | 1.00E-02 | Fitted |
| Dissolution shape | a.u. | 0.01 | 0.01 | 0.01 | 0.01 | 0.01 | 0.01 | 0.01 | Fitted |
| Dissolution time | min | 1000 | 1000 | 1000 | 1000 | 1000 | 1000 | 1000 | Fitted |
| Gestational Age | week | 2.00E+01 | 2.00E+01 | 2.00E+01 | 2.00E+01 | 2.00E+01 | 2.00E+01 | 4.00E+01 | Manual |
| Simulation Time to Steady-State | day | 5.00E+00 | 5.00E+00 | 4.00E+01 | 5.00E+00 | 4.00E+01 | 6.00E+01 | 6.00E+01 | Manual |
| CES1 Cytosol Vmax (Rat) | uM/min | 1.20E-01 | 1.20E-01 | 1.20E-01 | 1.20E-01 | 1.20E-01 | 1.20E-01 | 1.20E-01 | Literature |
| CES1 Microsome Vmax (Rat) | uM/min | 1.00E-01 | 1.00E-01 | 1.00E-01 | 1.00E-01 | 1.00E-01 | 1.00E-01 | 1.00E-01 | Literature |
| CES1 Plasma Vmax (Rat) | uM/min | 6.60E-01 | 6.60E-01 | 6.60E-01 | 6.60E-01 | 6.60E-01 | 6.60E-01 | 6.60E-01 | Literature |
| CYP1A2 Liver Vmax (Rat) | uM/min | 1.09E+00 | 1.09E+00 | 1.09E+00 | 1.09E+00 | 1.09E+00 | 1.09E+00 | 1.09E+00 | Literature |
| CES1 Cytosol Km (Rat) | uM | 9.30E-01 | 9.30E-01 | 9.30E-01 | 9.30E-01 | 9.30E-01 | 9.30E-01 | 9.30E-01 | Literature |
| CES1 Microsome Km (Rat) | uM | 7.60E-01 | 7.60E-01 | 7.60E-01 | 7.60E-01 | 7.60E-01 | 7.60E-01 | 7.60E-01 | Literature |
| CES1 Plasma Km (Rat) | uM | 1.79E+00 | 1.79E+00 | 1.79E+00 | 1.79E+00 | 1.79E+00 | 1.79E+00 | 1.79E+00 | Literature |
| CYP1A2 Liver Km (Rat) | uM | 7.60E-01 | 7.60E-01 | 7.60E-01 | 7.60E-01 | 7.60E-01 | 7.60E-01 | 7.60E-01 | Literature |
| CES1 Cytosol Vmax (Human) | uM/min | 1.38E+02 | 1.38E+02 | 1.38E+02 | 1.38E+02 | 1.38E+02 | 1.38E+02 | 1.38E+02 | Literature |
| CES1 Microsome Vmax (Human) | uM/min | 3.65E+02 | 3.65E+02 | 3.65E+02 | 3.65E+02 | 3.65E+02 | 3.65E+02 | 3.65E+02 | Literature |
| CES1 Cytosol Km (Human) | uM | 1.18E+00 | 1.18E+00 | 1.18E+00 | 1.18E+00 | 1.18E+00 | 1.18E+00 | 1.18E+00 | Literature |
| CES1 Microsome Km (Human) | uM | 3.81E+00 | 3.81E+00 | 3.81E+00 | 3.81E+00 | 3.81E+00 | 3.81E+00 | 3.81E+00 | Literature |

Table S4: Overview of human fetal/maternal plasma ratios and physicochemical properties of 109 investigated drugs.

| Drug | median | MW | LogP or LogD | Maternal Fu | Reference |
| --- | --- | --- | --- | --- | --- |
| Abacavir | 1.03 | 286.33 | 1.20 | 0.50 | (Chappuy *et al.*, 2004) |
| Acetylsalicylic acid | 0.40 | 180.16 | 1.19 | 0.01 | (Wolff *et al.*, 1982) |
| Acyclovir | 1.07 | 225.20 | -1.56 | 0.85 | (Haddad *et al.*, 1993) |
| Acyclovir | N/A | 225.20 | -1.56 | 0.85 | (Kimberlin *et al.*, 1998) |
| Alfentanil | 0.29 | 416.52 | 2.16 | 0.08 | (Gepts *et al.*, 1986) |
| Alfentanil | 0.29 | 416.52 | 2.16 | 0.08 | (Meuldermans *et al.*, 1986) |
| Ampicillin | 1.00 | 349.41 | 1.35 | 0.82 | (Maberry *et al.*, 1992) |
| Amprenavir | 0.27 | 505.63 | 1.85 | 0.10 | (Chappuy *et al.*, 2004) |
| Atazanavir | 0.13 | 704.86 | 4.08 | 0.14 | (Ripamonti *et al.*, 2007) |
| Atenolol | 0.81 | 266.34 | -1.29 | 0.89 | (Melander *et al.*, 1978) |
| Atenolol | 1.01 | 266.34 | -1.29 | 0.89 | (Hurst *et al.*, 1998) |
| Atropine sulphate | 0.66 | 676.82 | 1.83 | 0.74 | (Onnen *et al.*, 1979) |
| Betaxolol | 0.97 | 307.43 | 2.59 | 0.54 | (Morselli *et al.*, 1990) |
| Bupivacaine | 0.30 | 288.43 | 3.41 | 0.04 | (Reynolds and Taylor, 1970) |
| Bupivacaine | N/A | 288.43 | 3.41 | 0.04 | (Irestedt *et al.*, 1998) |
| Bupivacaine | N/A | 288.43 | 3.41 | 0.04 | (Ala-Kokko *et al.*, 1997) |
| Bupivacaine | N/A | 288.43 | 3.41 | 0.04 | (Abboud *et al.*, 1983) |
| Buprenorphine | 0.14 | 467.64 | 3.93 | 0.04 | (Concheiro *et al.*, 2011) |
| Buprenorphine | 0.35 | 467.64 | 3.93 | 0.04 | (Gordon *et al.*, 2010) |
| Carbamazepine | 0.77 | 236.27 | 2.45 | 0.26 | (Takeda *et al.*, 1992) |
| Carbamazepine | 0.80 | 236.27 | 2.45 | 0.26 | (Koristkova *et al.*, 2019) |
| Cefatrizine | 0.28 | 462.51 | N/A | N/A | (Bernard *et al.*, 1977) |
| Cefazolin | 0.18 | 476.50 | -1.89 | 0.11 | (Young *et al.*, 2015) |
| Cefazolin | 0.22 | 476.50 | -1.89 | 0.11 | (Young *et al.*, 2015) |
| Cefotaxime | 0.97 | 455.47 | -0.50 | 0.64 | (Maberry *et al.*, 1992) |
| Cefradine | 0.29 | 349.41 | -1.15 | 0.88 | (Craft and Forster, 1978) |
| Cefuroxime | N/A | 424.39 | -0.16 | 0.67 | (Craft *et al.*, 1981) |
| Cefuroxime | N/A | 424.39 | -0.16 | 0.67 | (Craft *et al.*, 1981) |
| Celiprolol | 0.25 | 414.00 | 0.13 | 0.71 | (Kofahl *et al.*, 1993) |
| Cimetidine | 0.33 | 252.34 | 0.40 | 0.91 | (McGowan, 1979) |
| Cimetidine | N/A | 252.34 | 0.40 | 0.91 | (Cimetidine in elective Caesarean section - Mccaughey - 1981 - Anaesthesia - Wiley Online Library) |
| Citalopram | 0.62 | 324.39 | 3.50 | 0.20 | (Hendrick *et al.*, 2003) |
| Citalopram | 0.83 | 324.39 | 3.50 | 0.20 | (Rampono *et al.*, 2009) |
| Clavulanic acid | 0.80 | 199.16 | -1.50 | 0.78 | (Maberry *et al.*, 1992) |
| Clomipramine | 0.40 | 314.85 | 5.19 | 0.03 | (Loughhead *et al.*, 2006) |
| Clonidine | N/A | 230.09 | 1.59 | 0.67 | (Buchanan *et al.*, 2009) |
| Clonidine | N/A | 230.09 | 1.59 | 0.67 | (Cigarini *et al.*, 1995) |
| Cortisol | 0.18 | 362.46 | 1.55 | 0.05 | (Petersen *et al.*, 1984) |
| Diazepam | 1.20 | 284.74 | 2.99 | 0.02 | (Moore and McBride, 1978) |
| Didanosine | 0.38 | 236.23 | -1.24 | 0.05 | (Chappuy *et al.*, 2004) |
| Digoxin | 0.26 | 780.94 | 1.26 | 0.77 | (Kerenyi *et al.*, 1980) |
| Digoxin | 0.90 | 780.94 | 1.26 | 0.77 | (Saarikoski, 1976) |
| Digoxin | 1.08 | 780.94 | 1.26 | 0.77 | (Ebara *et al.*, 1986) |
| Duloxetine | 0.12 | 297.42 | 4.72 | 0.10 | (Boyce *et al.*, 2011) |
| Efavirenz | 0.49 | 315.68 | 5.40 | 0.03 | (Cressey *et al.*, 2012) |
| Emtricitabine | 1.20 | 247.00 | -0.43 | 0.96 | (Stek *et al.*, 2012) |
| Emtricitabine | 1.63 | 247.00 | -0.43 | 0.96 | (Colbers *et al.*, 2013) |
| Enflurane | N/A | 184.49 | 2.10 | 0.03 | (Abboud *et al.*, 1985) |
| Enflurane | N/A | 184.49 | 2.10 | 0.03 | (Abboud *et al.*, 1985) |
| Escitalopram | 0.86 | 324.39 | 3.50 | 0.44 | (Rampono *et al.*, 2009) |
| Etidocaine | N/A | 276.42 | 3.66 | 0.07 | (Disposition and placental transfer of etidocaine in pregnancy - PubMed) |
| Famotidine | 1.37 | 337.45 | -0.64 | 0.83 | (Wang *et al.*, 2013) |
| Famotidine | 2.99 | 337.45 | -0.64 | 0.83 | (Wang *et al.*, 2013) |
| Fluoxetine | 0.54 | 309.33 | 4.05 | 0.06 | (Hendrick *et al.*, 2003) |
| Fluoxetine | 0.61 | 309.33 | 4.05 | 0.06 | (Rampono *et al.*, 2004) |
| Fluoxetine | 0.71 | 309.33 | 4.05 | 0.06 | (Rampono *et al.*, 2009) |
| Fluoxetine | 1.00 | 309.33 | 4.05 | 0.06 | (Kim *et al.*, 2000) |
| Fluoxetine | N/A | 309.33 | 4.05 | 0.06 | (Choi-Kwon *et al.*, 2006) |
| Fluoxetine, R-isomer | 0.80 | 309.33 | 4.05 | N/A | (Kim *et al.*, 2000) |
| Fluoxetine, S-isomer | 1.06 | 309.33 | 4.05 | N/A | (Kim *et al.*, 2000) |
| Fluvoxamine | 0.78 | 318.33 | 2.89 | 0.23 | (Rampono *et al.*, 2009) |
| Furosemide | 0.96 | 330.74 | 2.03 | 0.02 | (Riva *et al.*, 1978) |
| Gabapentin | 1.80 | 171.24 | -1.10 | 1.00 | (Ohman *et al.*, 2005) |
| Haloperidol | 0.69 | 375.86 | 4.30 | 0.08 | (Newport *et al.*, 2007) |
| Halothane | N/A | 197.38 | 2.30 | N/A | (Abboud *et al.*, 1985) |
| Hydroxyprogesterone caproate | 0.21 | 428.60 | 5.70 | N/A | (Caritis *et al.*, 2012) |
| Imipenem | 0.32 | 299.35 | -0.19 | 0.80 | (Pharmacokinetics and transplacental passage of imipenem during pregnancy - PubMed) |
| Indinavir | 0.01 | 613.79 | 2.90 | 0.40 | (Chappuy *et al.*, 2004) |
| Indomethacin | 5.06 | 357.79 | 4.27 | 0.03 | (Rytting *et al.*, 2014) |
| Labetalol | N/A | 328.41 | 1.24 | 0.50 | (Michael, 1979) |
| Lamivudine | 0.93 | 229.00 | -0.70 | 0.84 | (Chappuy *et al.*, 2004) |
| Lamivudine | 1.06 | 229.00 | -0.70 | 0.84 | (Mandelbrot *et al.*, 2001) |
| Lamivudine | 1.12 | 229.00 | -0.70 | 0.84 | (Moodley *et al.*, 2001) |
| Lamotrigine | 0.91 | 256.09 | 1.19 | 0.35 | (Kacirova *et al.*, 2010) |
| Lamotrigine | 1.00 | 256.09 | 1.19 | 0.35 | (Ohman *et al.*, 2000) |
| Lamotrigine | 1.02 | 256.09 | 1.19 | 0.35 | (Myllynen *et al.*, 2003) |
| Lamotrigine | 1.21 | 256.09 | 1.19 | 0.35 | (Tomson *et al.*, 1997) |
| Lamotrigine | 1.55 | 256.09 | 1.19 | 0.35 | (Myllynen *et al.*, 2003) |
| Lamotrigine | N/A | 256.09 | 1.19 | 0.35 | (de Haan *et al.*, 2004) |
| Lidocaine | 0.54 | 234.34 | 2.44 | 0.38 | (Shnider and Way, 1968) |
| Lidocaine | 0.59 | 234.34 | 2.44 | 0.38 | (Fox and Houle, 1969) |
| Lopinavir | 0.20 | 628.80 | 3.91 | 0.83 | (Aweeka *et al.*, 2010) |
| Lopinavir | 0.23 | 628.80 | 3.91 | 0.83 | (Mirochnick *et al.*, 2008) |
| Lopinavir | 0.24 | 628.80 | 3.91 | 0.83 | (van Hoog *et al.*, 2012) |
| Mecillinam | N/A | 325.43 | -1.67 | 0.93 | (Heikkilä and Erkkola, 1994) |
| Meperidine | N/A | 247.33 | 2.72 | 0.70 | (Nation, 1981) |
| Metformin | 0.70 | 129.16 | -1.43 | 1.00 | (de Oliveira Baraldi *et al.*, 2011) |
| Metformin | 1.64 | 129.16 | -1.43 | 1.00 | (Vanky *et al.*, 2005) |
| Metoprolol, R-isomer | 1.04 | 267.36 | -0.16 | 0.80 | (Antunes *et al.*, 2015) |
| Metoprolol, S-isomer | 1.15 | 267.36 | -0.16 | 0.81 | (Antunes *et al.*, 2015) |
| Metronidazole | 0.87 | 171.15 | -0.14 | 0.85 | (Visser and Hundt, 1984) |
| Nelfinavir | 0.14 | 567.78 | 4.61 | 0.02 | (van Hoog *et al.*, 2012) |
| Nelfinavir | 0.24 | 567.78 | 4.61 | 0.02 | (Chappuy *et al.*, 2004) |
| Nelfinavir | 0.25 | 567.78 | 4.61 | 0.02 | (Hirt *et al.*, 2007) |
| Nelfinavir | N/A | 567.78 | 4.61 | 0.02 | (Gingelmaier *et al.*, 2006) |
| Nevirapine | 0.67 | 266.30 | 1.20 | 0.40 | (van Hoog *et al.*, 2012) |
| Nevirapine | N/A | 266.30 | 1.20 | 0.40 | (Gingelmaier *et al.*, 2006) |
| Nicardipine | 0.17 | 479.53 | 3.82 | 0.01 | (Bartels *et al.*, 2007) |
| Nifedipine | 0.64 | 346.33 | 2.20 | 0.06 | (Silberschmidt *et al.*, 2008) |
| Nifedipine | N/A | 346.33 | 2.20 | 0.06 | (Pirhonen *et al.*, 1990) |
| Nifedipine | N/A | 346.33 | 2.20 | 0.06 | Prevost 1992 |
| Norbuprenorphine | 0.49 | 413.55 | 1.18 | N/A | Gordon 2010 |
| Norfluoxetine | 1.07 | 295.30 | 3.80 | N/A | (Kim *et al.*, 2000) |
| Norfluoxetine, R-isomer | 0.84 | 295.30 | 3.80 | N/A | (Kim *et al.*, 2000) |
| Norfluoxetine, S-isomer | 1.16 | 295.30 | 3.80 | N/A | (Kim *et al.*, 2000) |
| Nortriptyline | 0.68 | 263.38 | 4.51 | 0.06 | (Loughhead *et al.*, 2006) |
| Olanzapine | 0.32 | 312.43 | 2.00 | 0.07 | (Aichhorn *et al.*, 2008) |
| Olanzapine | 0.70 | 312.43 | 2.00 | 0.07 | (Newport *et al.*, 2007) |
| Oxcarbazepine | 0.11 | 252.27 | 1.76 | 0.41 | (Pienimäki *et al.*, 1997) |
| Oxcarbazepine | 0.25 | 252.27 | 1.76 | 0.41 | (Myllynen *et al.*, 2001) |
| Oxcarbazepine | 0.71 | 252.27 | 1.76 | 0.41 | (Myllynen *et al.*, 2001) |
| Oxcarbazepine | 0.76 | 252.27 | 1.76 | 0.41 | (Myllynen *et al.*, 2001) |
| Oxcarbazepine | 0.83 | 252.27 | 1.76 | 0.41 | (Myllynen *et al.*, 2001) |
| Oxcarbazepine | 2.75 | 252.27 | 1.76 | 0.41 | (Pienimäki *et al.*, 1997) |
| Oxcarbazepine | 2.83 | 252.27 | 1.76 | 0.41 | (Myllynen *et al.*, 2001) |
| Oxcarbazepine | 3.22 | 252.27 | 1.76 | 0.41 | (Pienimäki *et al.*, 1997) |
| Oxcarbazepine | 4.33 | 252.27 | 1.76 | 0.41 | (Myllynen *et al.*, 2001) |
| Oxprenolol | 0.29 | 265.35 | 2.18 | 0.30 | (Sioufi *et al.*, 1984) |
| Paracetamol | 0.78 | 151.16 | 0.78 | 1.00 | (Roberts *et al.*, 1984) |
| Paroxetine | 0.15 | 329.37 | 3.10 | 0.05 | (Rampono *et al.*, 2009) |
| Paroxetine | 0.50 | 329.37 | 3.10 | 0.05 | (Rampono *et al.*, 2004) |
| Paroxetine | 0.56 | 329.37 | 3.10 | 0.05 | (Hendrick *et al.*, 2003) |
| Phenobarbital | 0.92 | 232.24 | 1.47 | 0.56 | (Nau *et al.*, 1980) |
| Phenobarbital | N/A | 232.24 | 1.47 | 0.56 | (Takeda *et al.*, 1992) |
| Phenylethylmalonamide | 0.94 | 206.24 | N/A | N/A | (Nau *et al.*, 1980) |
| Phenytoin | 0.89 | 252.27 | 2.47 | 0.10 | (Takeda *et al.*, 1992) |
| Phenytoin | 0.90 | 252.27 | 2.47 | 0.10 | (Mirkin, 1971) |
| Phenytoin | 0.96 | 252.27 | 2.47 | 0.10 | (Mirkin, 1971) |
| Phenytoin | 1.22 | 252.27 | 2.47 | 0.10 | (Mirkin, 1971) |
| Pindolol, R-isoform | 0.60 | 248.32 | 1.75 | 0.51 | (Gonçalves *et al.*, 2007) |
| Pindolol, S-isoform | 0.70 | 248.32 | 1.75 | 0.76 | (Gonçalves *et al.*, 2007) |
| Piperacillin | 0.27 | 517.56 | 0.67 | 0.70 | (Heikkilä and Erkkola, 1994) |
| Prazosin | 0.09 | 383.40 | 1.88 | 0.06 | (Bourget *et al.*, 1995) |
| Prazosin | 0.20 | 383.40 | 1.88 | 0.06 | (Bourget *et al.*, 1995) |
| Prazosin | 0.23 | 383.40 | 1.88 | 0.06 | (Bourget *et al.*, 1995) |
| Prednisolone | 0.12 | 360.44 | 1.62 | 0.54 | (van Runnard Heimel *et al.*, 2005) |
| Prednisone | 1.67 | 358.43 | 1.46 | 0.18 | (van Runnard Heimel *et al.*, 2005) |
| Primidone | 0.93 | 218.25 | 0.91 | 0.30 | (Nau *et al.*, 1980) |
| Propofol | 0.62 | 178.27 | 3.79 | 0.99 | (Dailland *et al.*, 1989) |
| Propofol | 0.70 | 178.27 | 3.79 | 0.99 | (Dailland *et al.*, 1989) |
| Propranolol | 0.28 | 259.34 | 3.48 | 0.07 | (Erkkola *et al.*, 1982) |
| Quetiapine | 0.21 | 383.51 | 2.93 | 0.17 | (Newport *et al.*, 2007) |
| Risperidone | 0.42 | 410.48 | 3.04 | 0.16 | (Newport *et al.*, 2007) |
| Ritodrine | 0.21 | 287.35 | 1.22 | 0.46 | (Fujimoto *et al.*, 1986) |
| Ritodrine | 0.31 | 287.35 | 1.22 | 0.46 | (Lierde and Thomas, 1982) |
| Ritodrine | 0.56 | 287.35 | 1.22 | 0.46 | (Gandar *et al.*, 1980) |
| Ritodrine | 0.67 | 287.35 | 1.22 | 0.46 | (Fujimoto *et al.*, 1991) |
| Ritodrine | 0.75 | 287.35 | 1.22 | 0.46 | (Fujimoto *et al.*, 1991) |
| Ritodrine | 0.96 | 287.35 | 1.22 | 0.46 | (Gross *et al.*, 1985) |
| Ritonavir | 0.00 | 720.94 | 4.30 | 0.45 | (Chappuy *et al.*, 2004) |
| Salbutamol | 0.70 | 239.31 | 0.44 | 0.92 | (Boulton and Fawcett, 1997) |
| Salicylic acid | 0.74 | 138.12 | -1.33 | 0.05 | (Wolff *et al.*, 1982) |
| Saquinavir | 0.00 | 670.84 | 2.03 | 0.02 | (Chappuy *et al.*, 2004) |
| Sertraline | 0.27 | 306.23 | 5.06 | 0.02 | (Hendrick *et al.*, 2003) |
| Sertraline | 0.33 | 306.23 | 5.06 | 0.02 | (Rampono *et al.*, 2009) |
| Sertraline | 0.38 | 306.23 | 5.06 | 0.02 | (Rampono *et al.*, 2004) |
| Sertraline | 0.45 | 306.23 | 5.06 | 0.02 | (Rampono *et al.*, 2004) |
| Sertraline | 0.88 | 306.23 | 5.06 | 0.02 | (Rampono *et al.*, 2004) |
| Sertraline | 1.20 | 306.23 | 5.06 | 0.02 | (Rampono *et al.*, 2004) |
| Sotalol | 0.47 | 272.36 | -1.35 | 0.83 | (Erkkola *et al.*, 1982) |
| Stavudine | 1.32 | 224.21 | -0.72 | 1.00 | (Chappuy *et al.*, 2004) |
| Sulbactam | 1.30 | 233.24 | N/A | N/A | (Maberry *et al.*, 1992) |
| Sulindac | 0.99 | 356.41 | 3.42 | 0.07 | (Kramer *et al.*, 1995) |
| Suphadoxine | 0.98 | 310.33 | 0.70 | 0.06 | (Trenque *et al.*, 1998) |
| Tenofovir | 0.82 | 287.00 | -2.21 | 0.99 | (Colbers *et al.*, 2013) |
| Tenofovir | 0.88 | 287.00 | -2.21 | 0.99 | (Best *et al.*, 2015) |
| Theophylline | 0.92 | 180.16 | -0.02 | 0.52 | (Arwood *et al.*, 1979) |
| Theophylline | 1.00 | 180.16 | -0.02 | 0.52 | (Arwood *et al.*, 1979) |
| Theophylline | 1.02 | 180.16 | -0.02 | 0.52 | (Labovitz and Spector, 1982) |
| Ticarcillin | 0.60 | 384.43 | 0.99 | 0.60 | (Maberry *et al.*, 1992) |
| Tobramycin | 1.68 | 467.51 | -3.00 | 1.00 | (Bourget *et al.*, 1991) |
| Topiramate | 0.96 | 339.36 | 0.57 | 0.87 | (Ohman *et al.*, 2002) |
| Valproic acid | 1.32 | 144.21 | 2.75 | 0.20 | (Kacirova *et al.*, 2015) |
| Valproic acid | 1.57 | 144.21 | 2.75 | 0.20 | (Takeda *et al.*, 1992) |
| Venlafaxine | 0.72 | 277.40 | 2.69 | 0.73 | (Rampono *et al.*, 2009) |
| Venlafaxine | 1.10 | 277.40 | 2.69 | 0.73 | (Rampono *et al.*, 2004) |
| Zidovudine | 1.13 | 267.24 | 0.05 | 0.73 | (Watts *et al.*, 1991) |
| Zidovudine | 1.22 | 267.24 | 0.05 | 0.73 | (Chappuy *et al.*, 2004) |
| Zidovudine | 1.24 | 267.24 | 0.05 | 0.73 | (Moodley *et al.*, 2001) |
| Zidovudine | 1.27 | 267.24 | 0.05 | 0.73 | (Watts *et al.*, 1991) |

# Sensitivity Analysis

We performed a sensitivity analysis on the final developed human maternal-fetal PBPK model to investigate the effect of changing model parameters on the maximum concentration in the maternal brain and the fetal plasma (which will directly impact the fetal brain concentration estimation). In a sensitivity analysis, model parameters are deviated (e.g., increased by 10%), and the effect on PK-related parameters (Cmax, Tmax, AUC, etc.) are investigated. Overall, model sensitivity across parameters was moderate. As can be seen in Figure 1, among the most impactful parameters to increase brain Cmax are the lipophilicity (100%), the brain partition coefficient (50%), the administered dose (100%), and metabolism-related parameters (e.g., if the carboxylesterase 1 (CES1) ontogeny factor would be increased by 10%, a 6% decrease in Cmax would be observed (sensitivity of ~60%)).


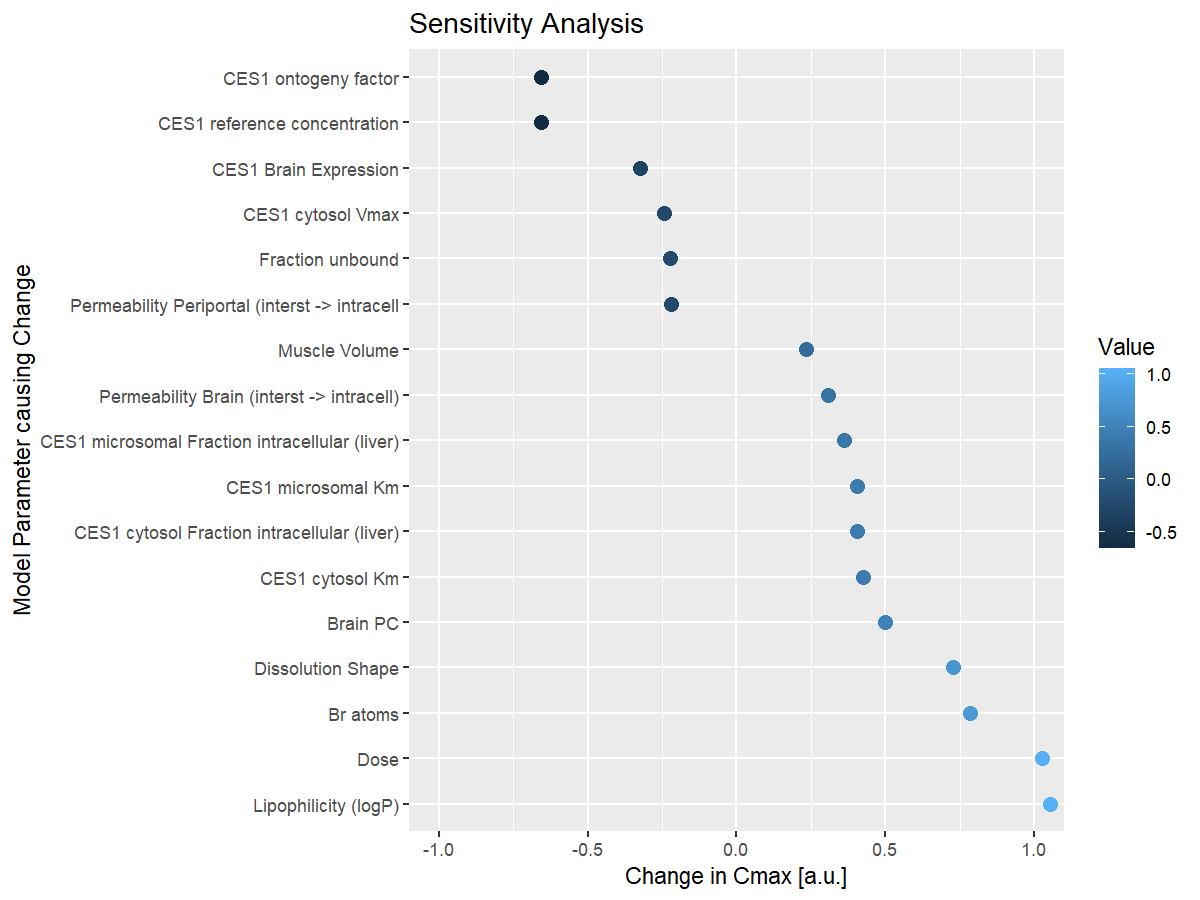


**Supp. Fig. 1:** Results of the sensitivity analysis, which investigates the effect of changing model parameters on PK-related outcome parameters. In this case, the effect of model parameters on the estimated maximum concentration in the maternal brain are presented.

Similarly, the parameters primarily affecting the fetal plasma maximum concentration can be seen in Figure 2. Here, dose (100%), placenta surface area (60%), placenta permeability (~60%) and the umbilical cord volume (-30%) are among the most impactful parameters to change fetal plasma maximum concentrations.


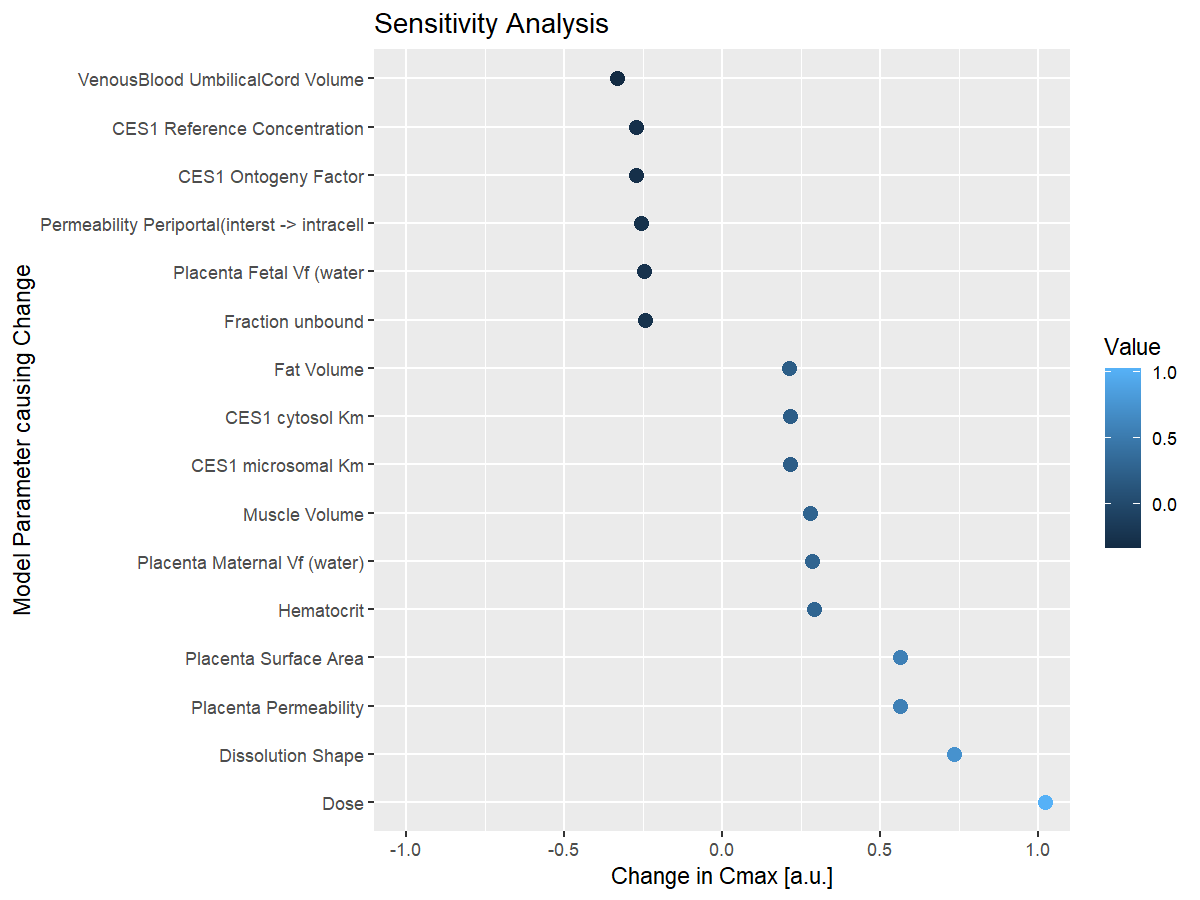


**Supp. Fig. 2:** Results of the sensitivity analysis for the change in fetal plasma maximum concentration. Only parameters causing more than -+2% change were included.

In summary, the sensitivity analysis highlights parameters that mainly affect, in this case, the concentration maximum simulated in a particular compartment (here: plasma or brain). Such an analysis allows setting the uncertainty of a specific model parameter into context with its impact on relevant PK-related parameters, such as concentration maximum. In an extreme case, for example, the fraction unbound could be 100% larger than currently implemented in PK-Sim (0.1 -> 0.2), we would then expect a 25% decrease in the simulated plasma concentrations in the fetus (Figure S2: shows a value of -25% for fraction unbound). The confidence interval for the fitted parameter values is small (results not shown). Thus, the confidence, that the identified parameter value is correct is large. Taken together, the sensitivity analysis, as well as the confidence intervals, confirm confidence in the results for the realistic data-driven approach.

##

# Further Simulation Results - Human Model

###
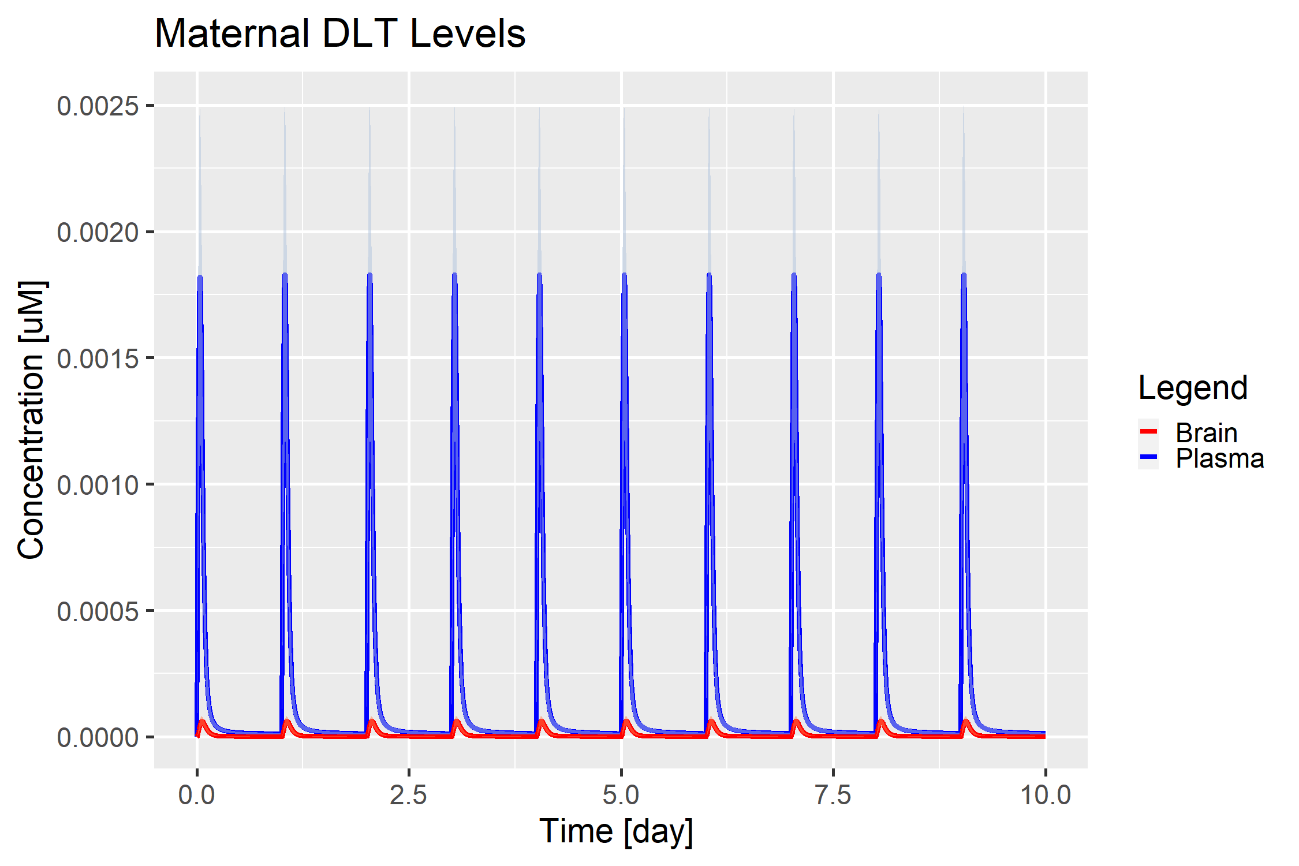


###
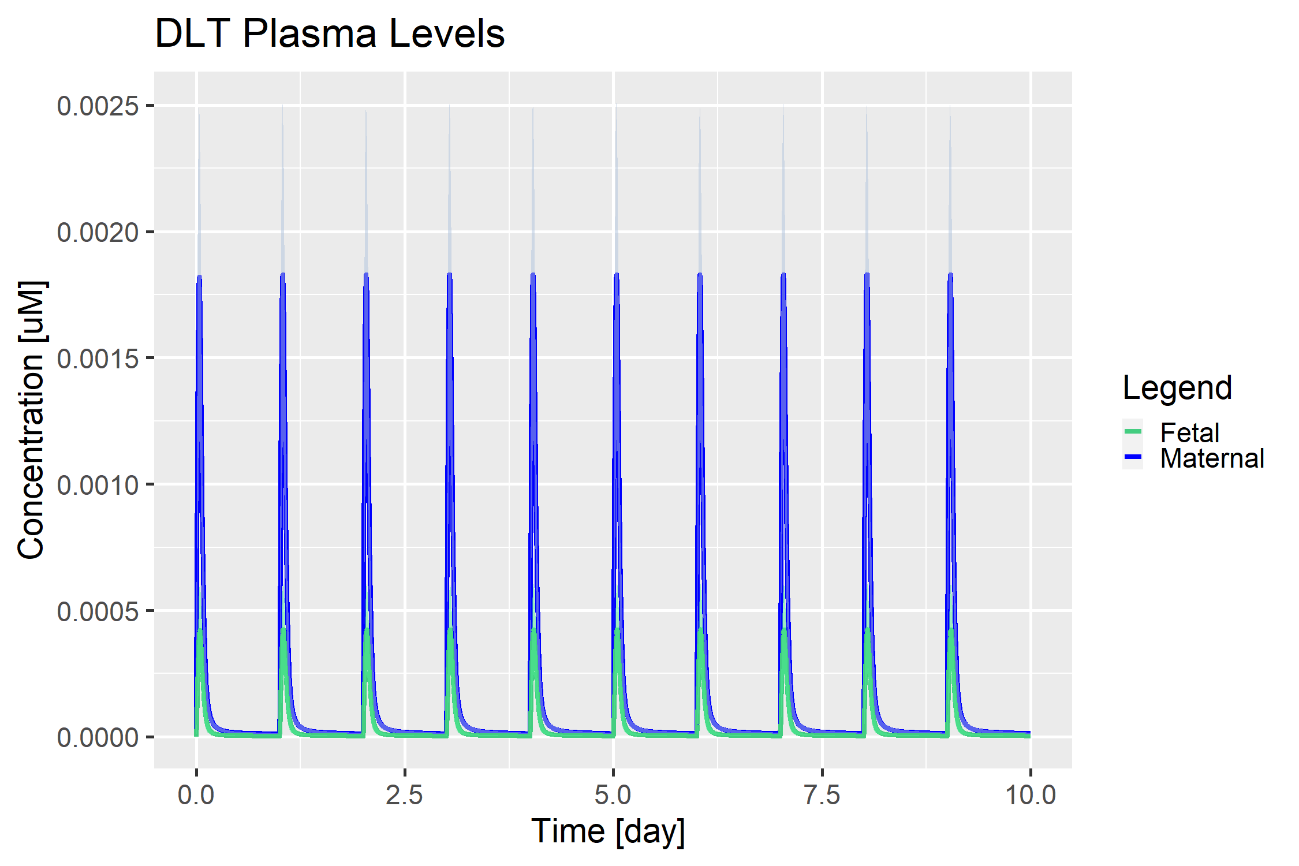


**Supp. Fig. 4.** Model simulations for Case 1 after single daily administrations of 0.01 mg/kg (ADI) for maternal brain (red), maternal plasma (blue), and fetal plasma (green). Solid lines represent the population means over 100 simulations, and shaded areas represent 1 standard deviation.

**
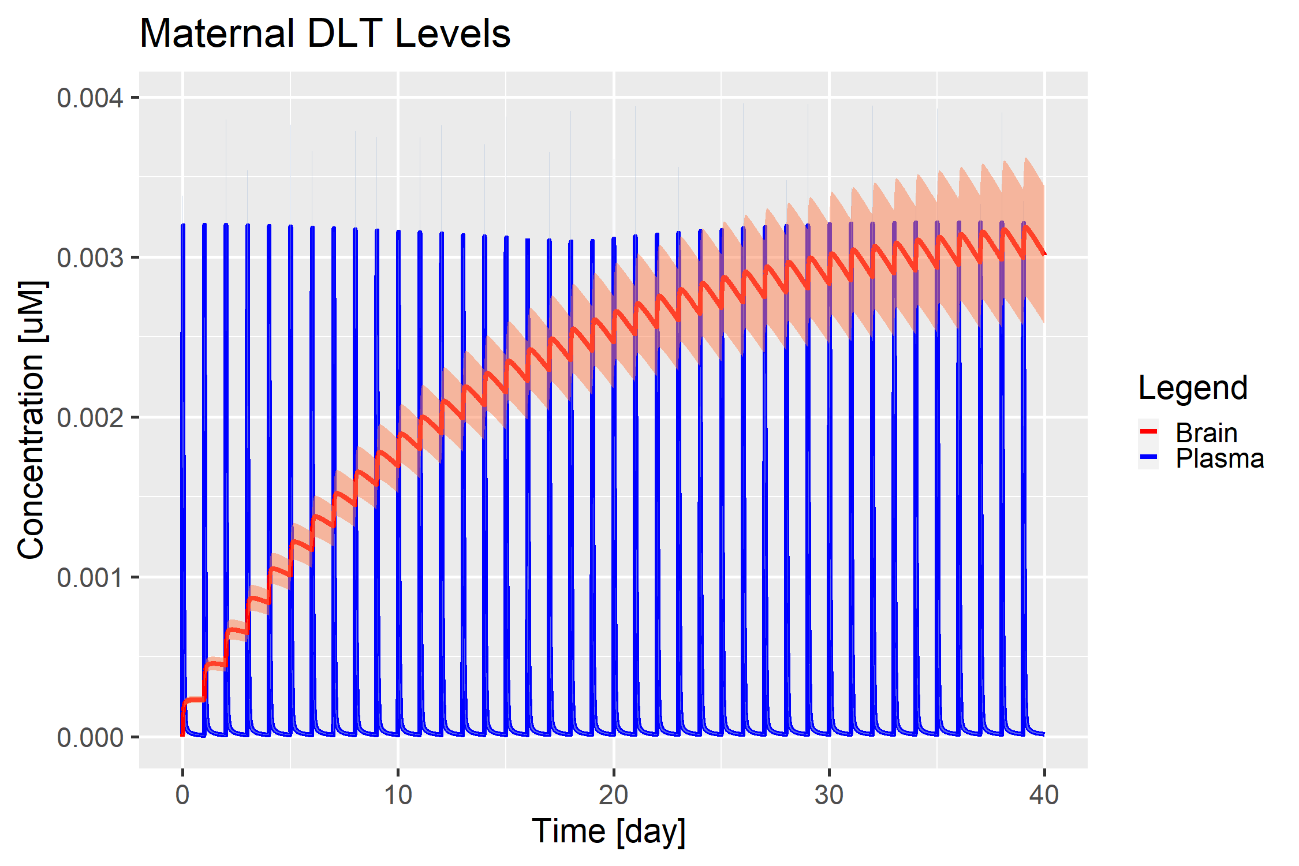
**

**
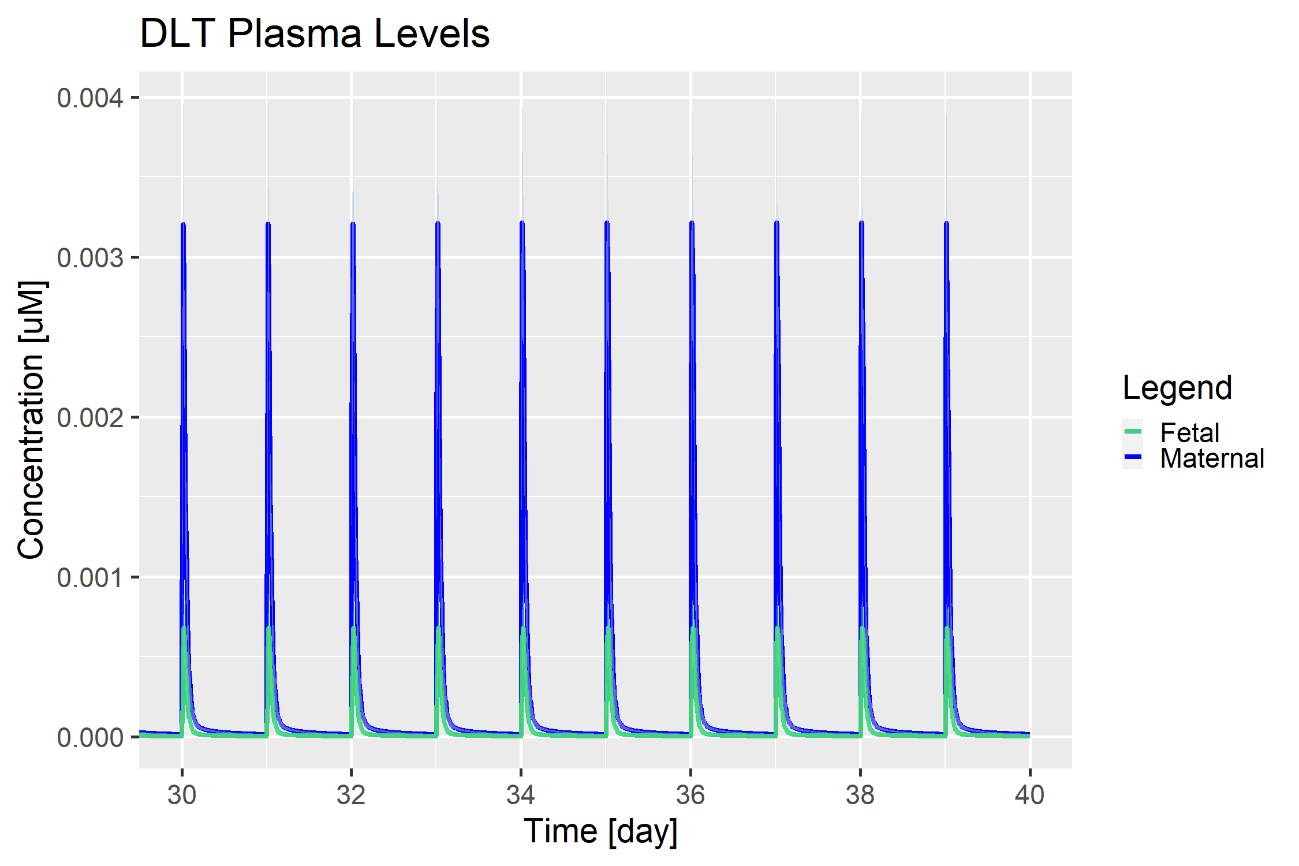
**

**Supp. Fig. 5.** Model simulations for Case 3 after single daily administrations of 0.01 mg/kg (ADI) for maternal brain (red), maternal plasma (blue), and fetal plasma (green). Solid lines represent the population means over 100 simulations, and shaded areas represent 1 standard deviation. The effect of an impaired maternal BBB is present (top) and shows an accumulation effect of DLT after ~ 40 days of repeated dosing.


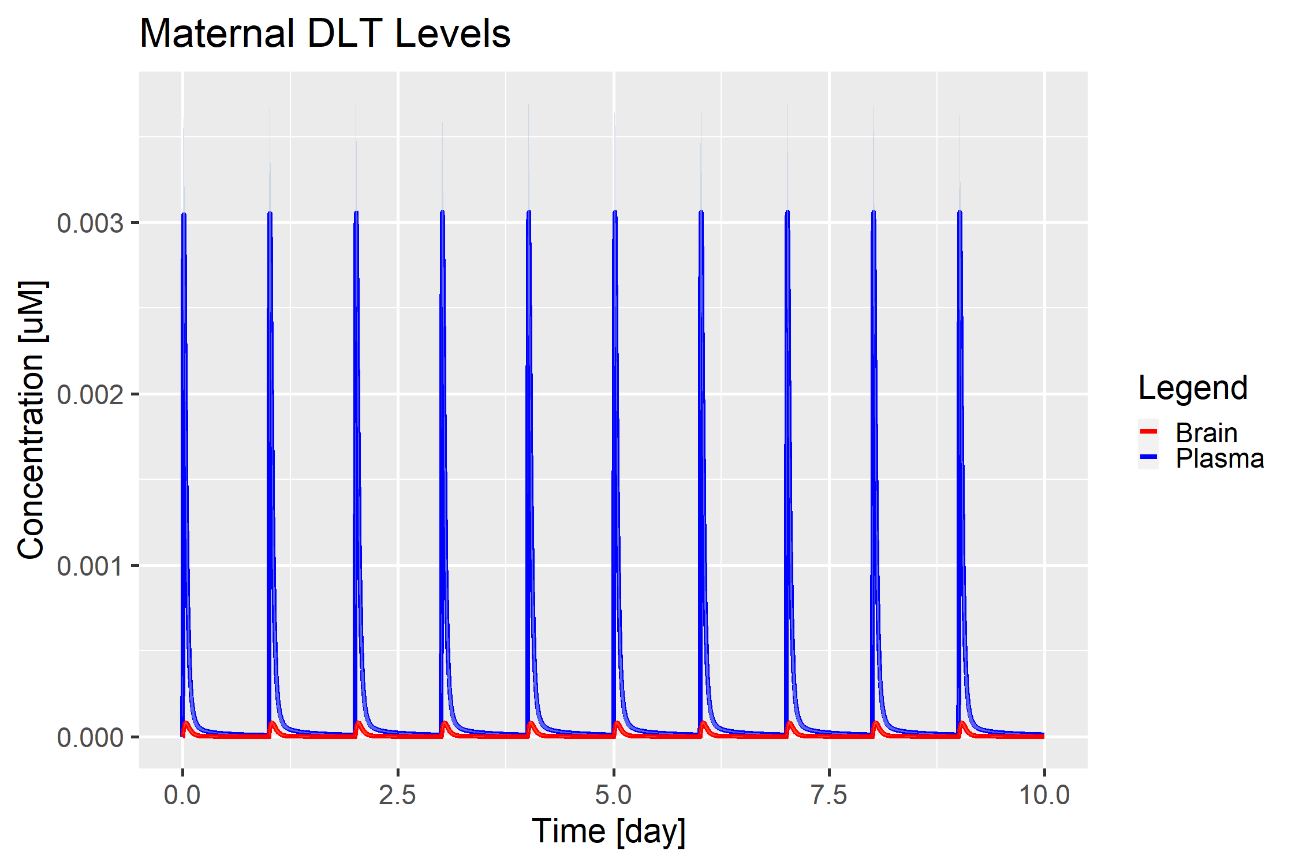

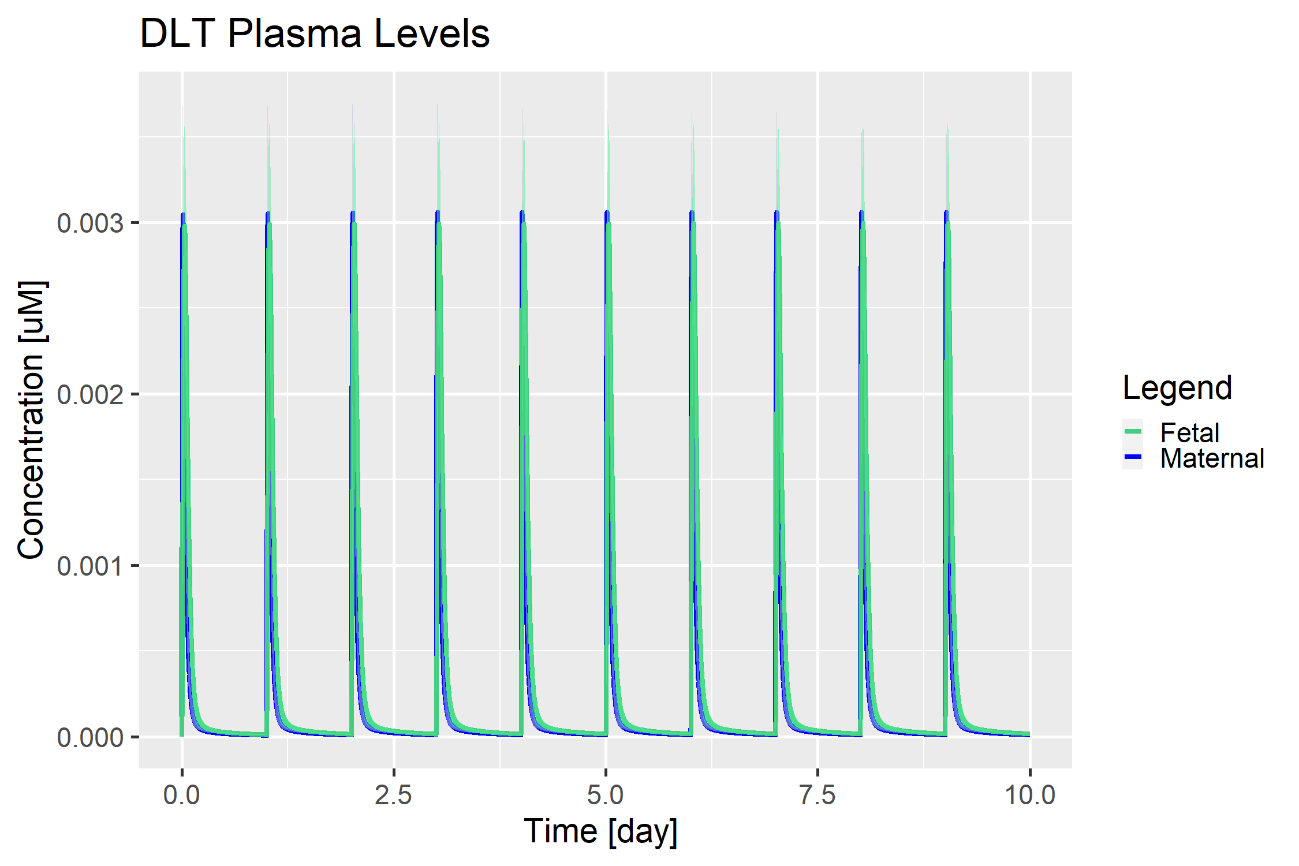


**Supp. Fig. 6.** Model simulations for Case 4 after single daily administrations of 0.01 mg/kg (ADI) for maternal brain (red), maternal plasma (blue), and fetal plasma (green). Solid lines represent the population means over 100 simulations, and shaded areas represent 1 standard deviation. The effect of an impaired BPB is apparent as both the fetal and maternal plasma kinetics (bottom) overlap.

**
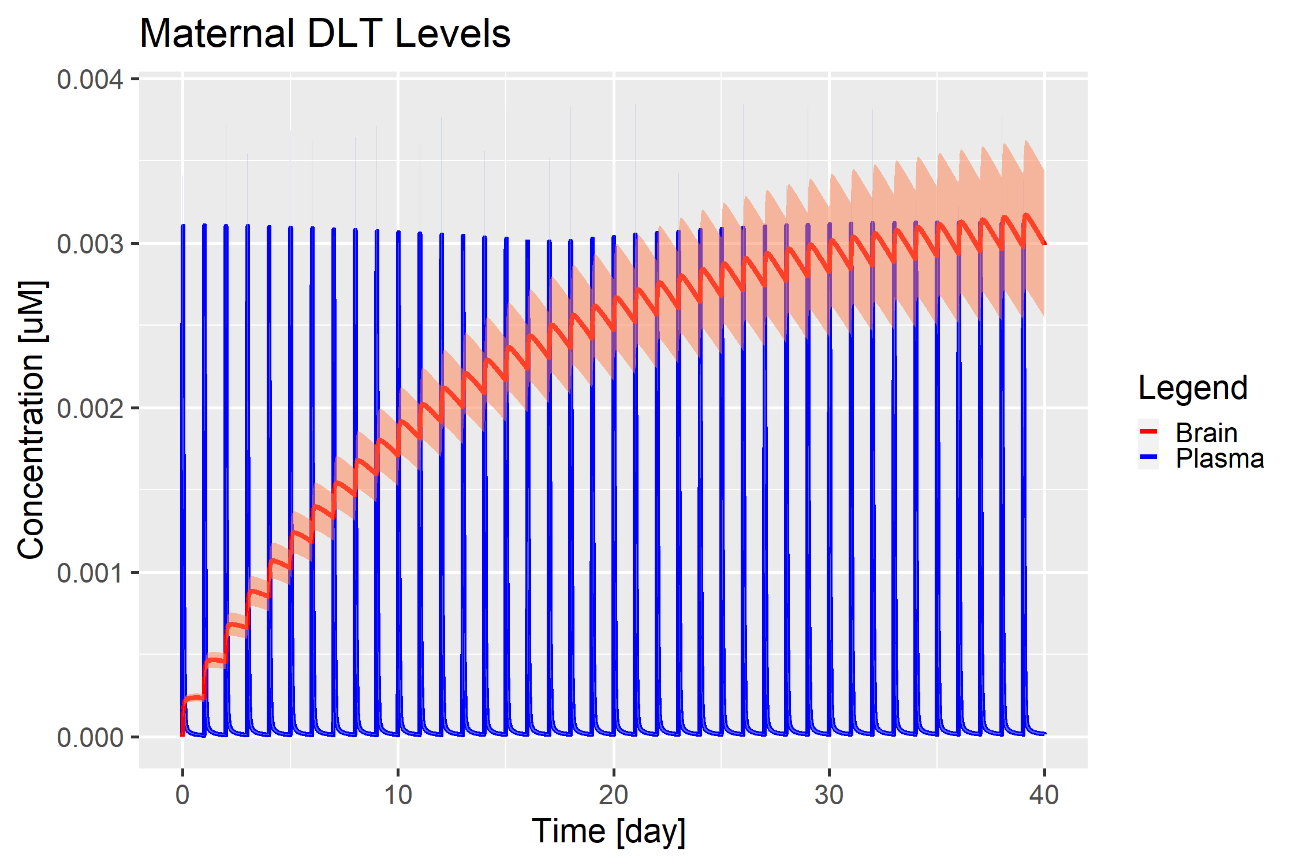
**

**
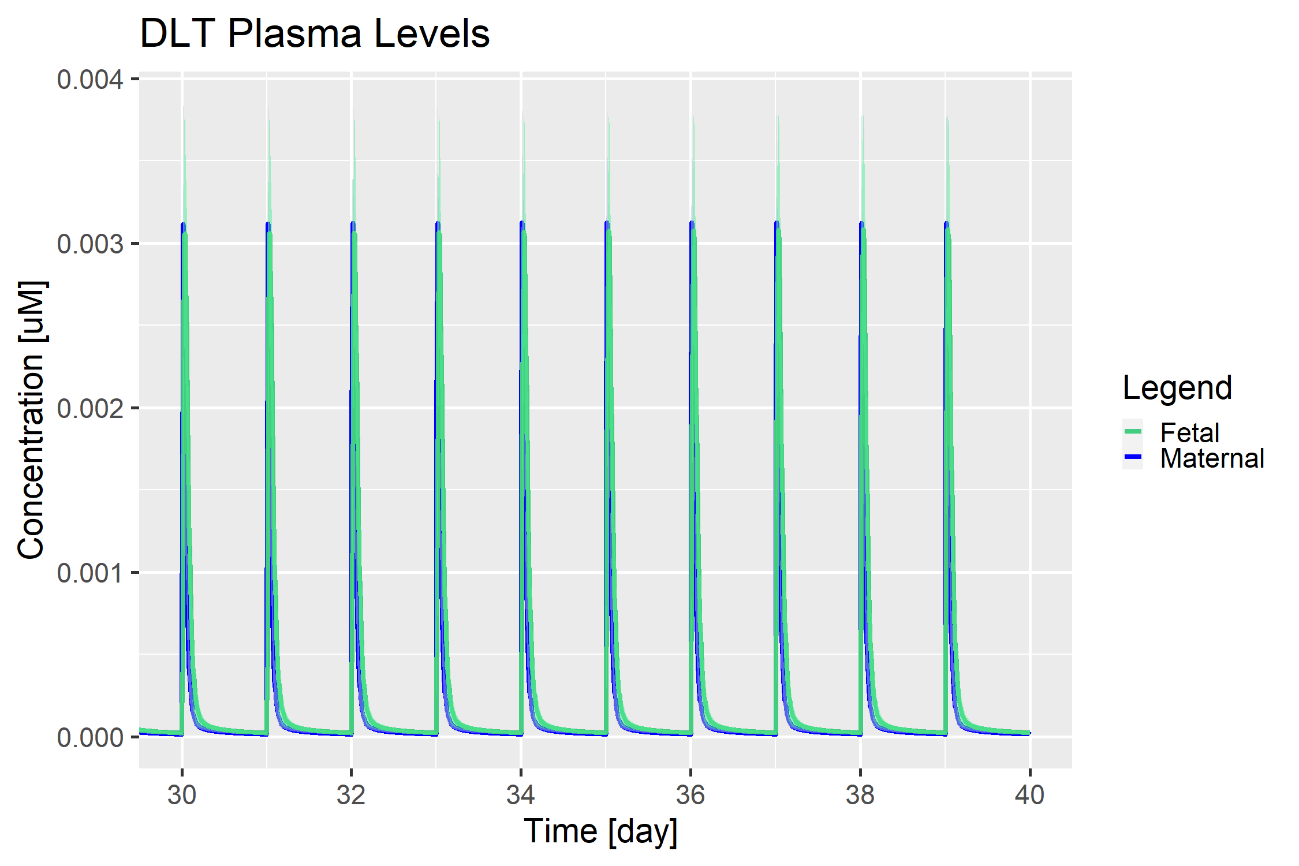
**

**Supp. Fig. 7.** Model simulations for Case 5 after single daily administrations of 0.01 mg/kg (ADI) for maternal brain (red), maternal plasma (blue), and fetal plasma (green). Solid lines represent the population means over 100 simulations, and shaded areas represent 1 standard deviation. In addition to Case 4, now the maternal BBB is also impaired, resulting in an accumulation of DLT in the maternal brain.

**
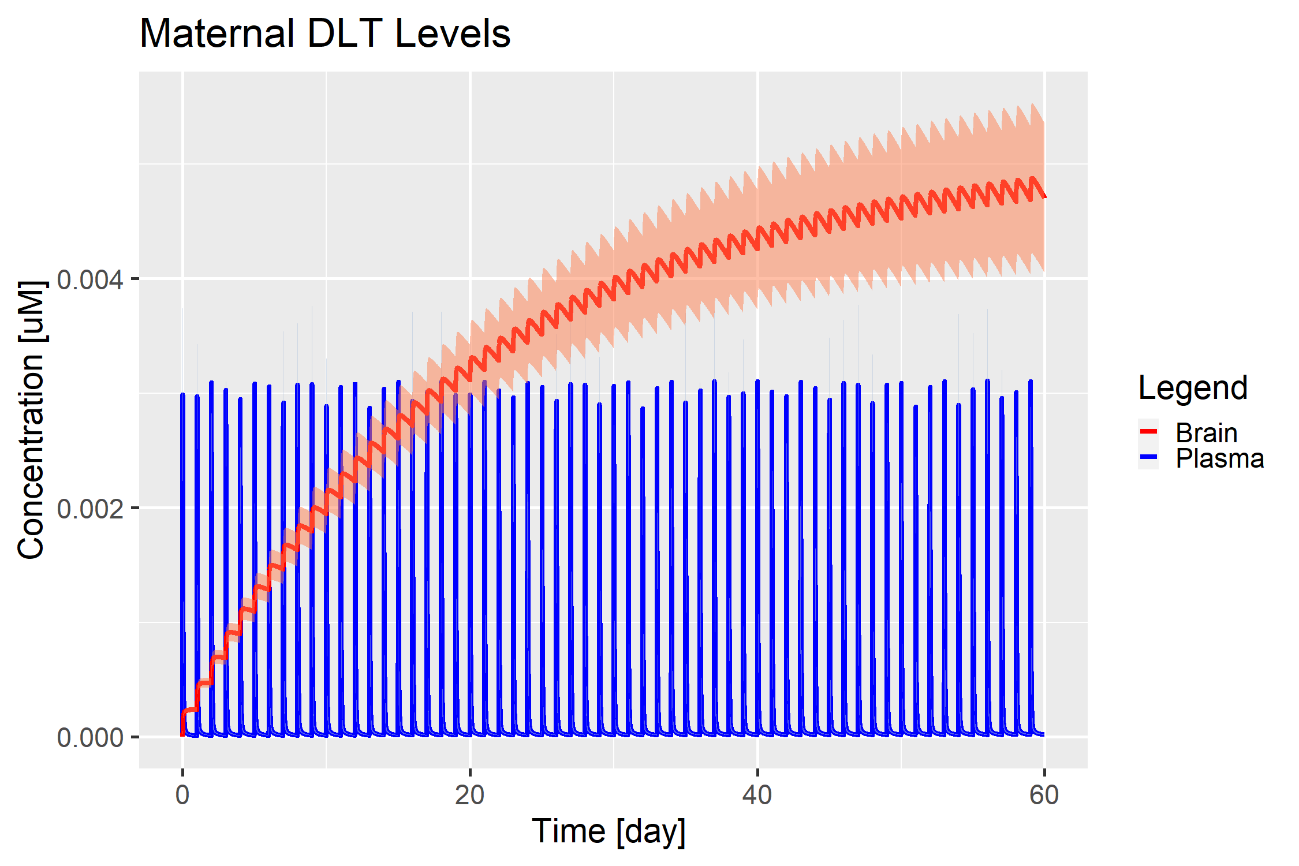
**

**
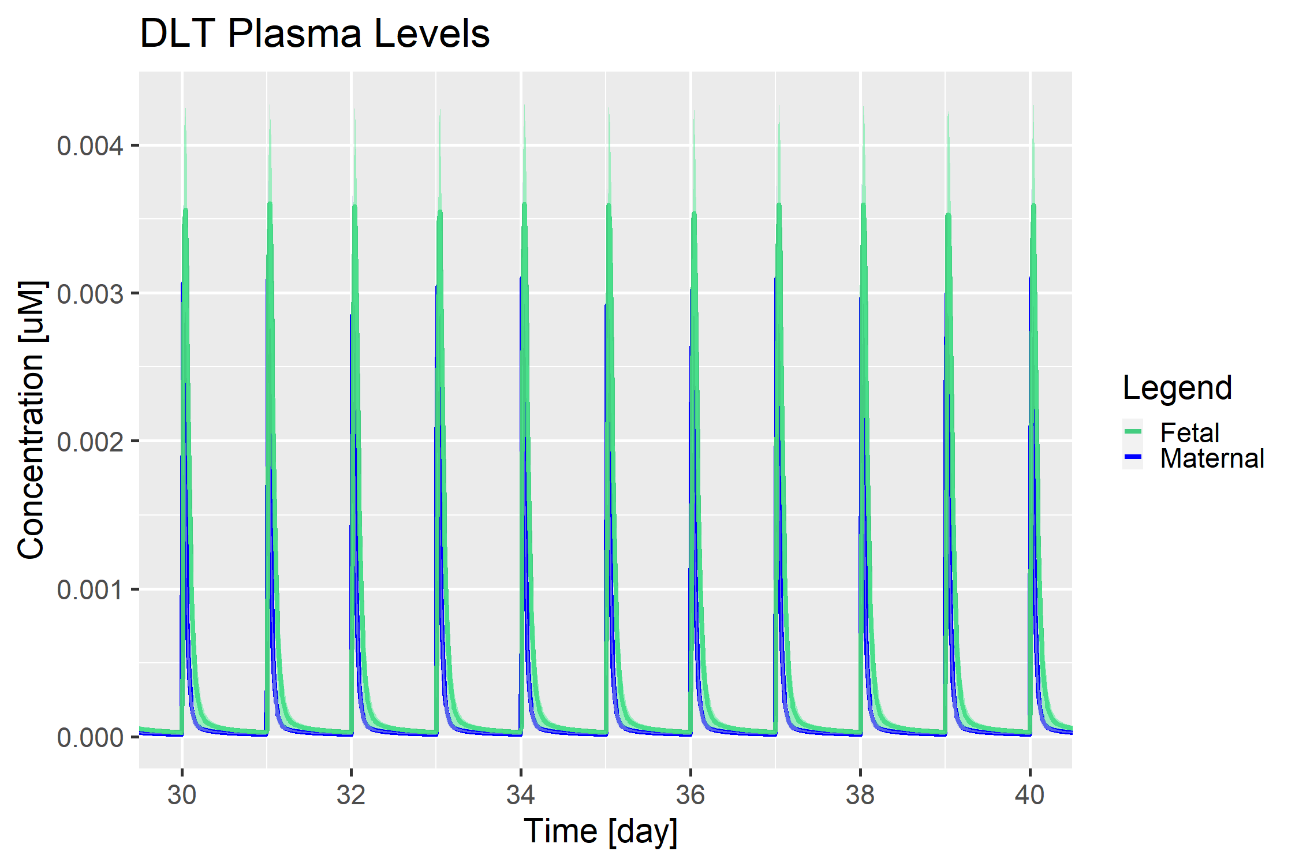
**

**Supp. Fig. 8.** Model simulations for Case 6 after single daily administrations of 0.01 mg/kg (ADI) for maternal brain (red), maternal plasma (blue), and fetal plasma (green). Solid lines represent the population means over 100 simulations, and shaded areas represent 1 standard deviation. The accumulation effect in the maternal brain is even more pronounced, while the fetal plasma concentration shows an increased ratio of 1.2 (zoomed into the last 10 days for graphical purposes only).

### Case 3


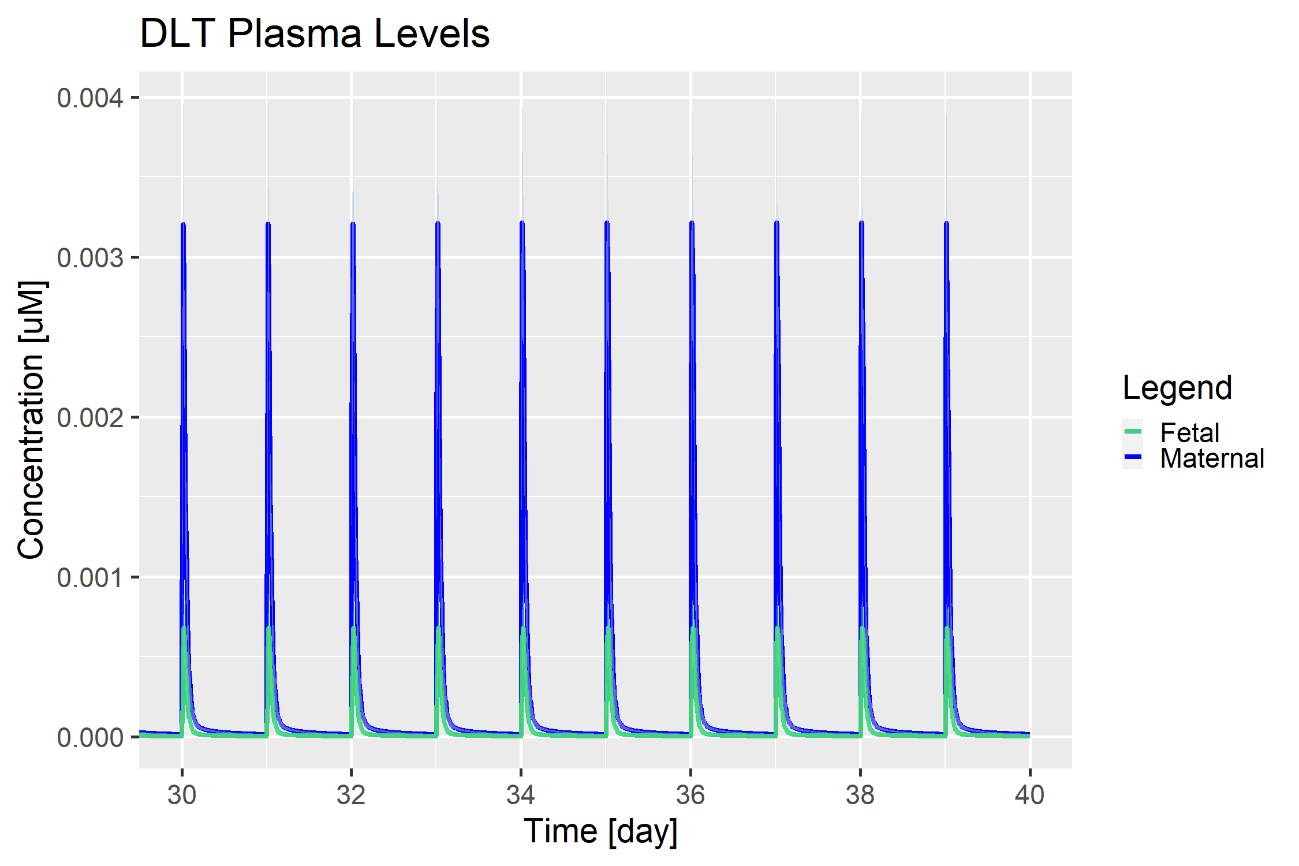


Case 5


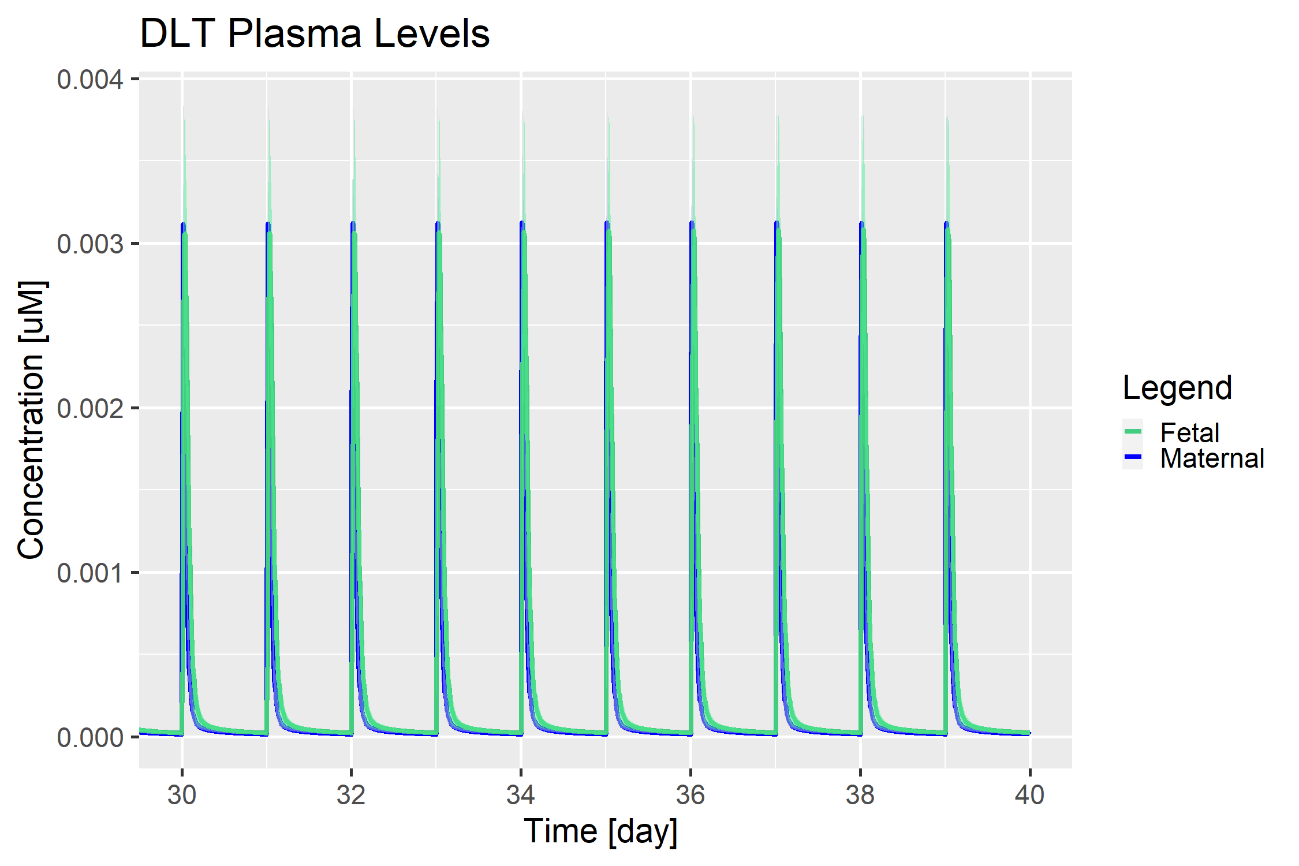


### Case 6


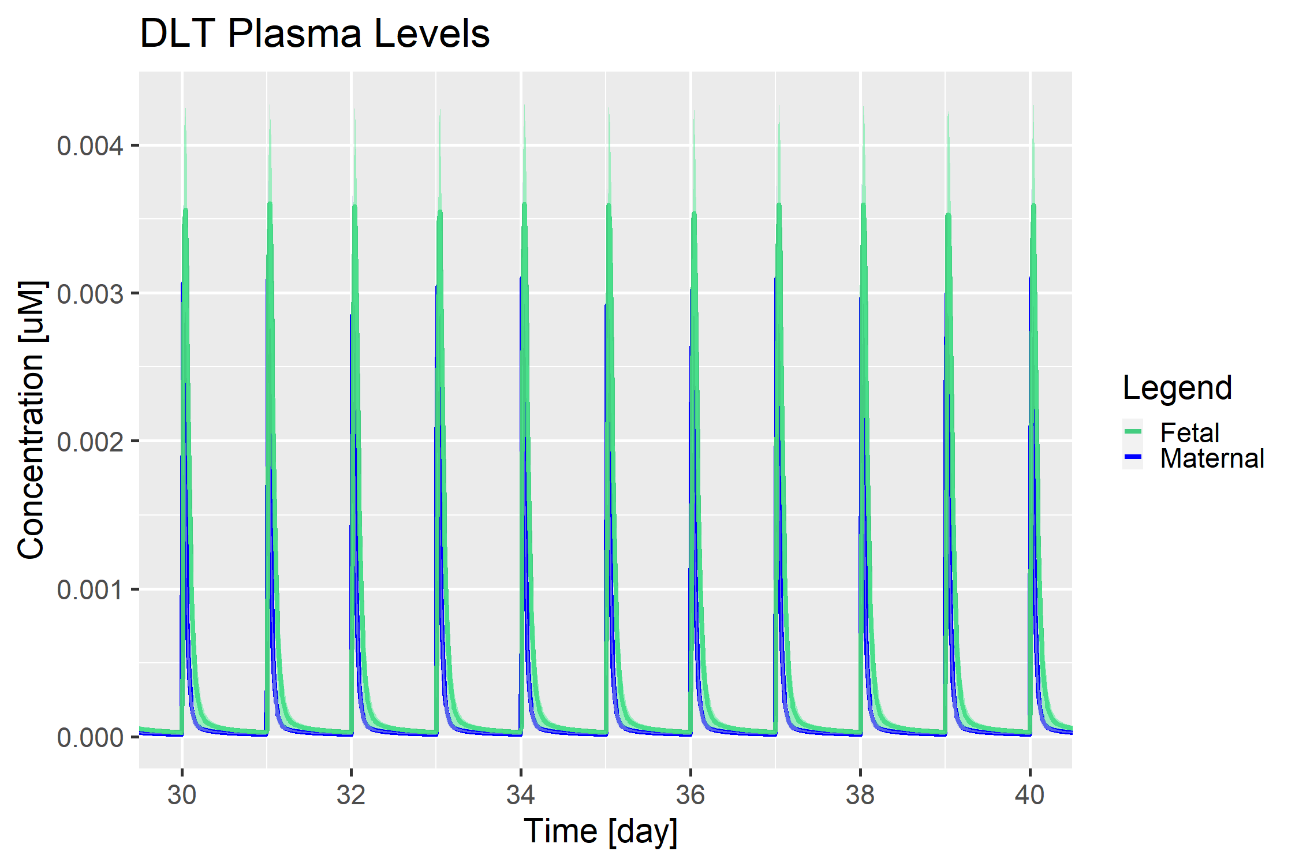


### Case 7


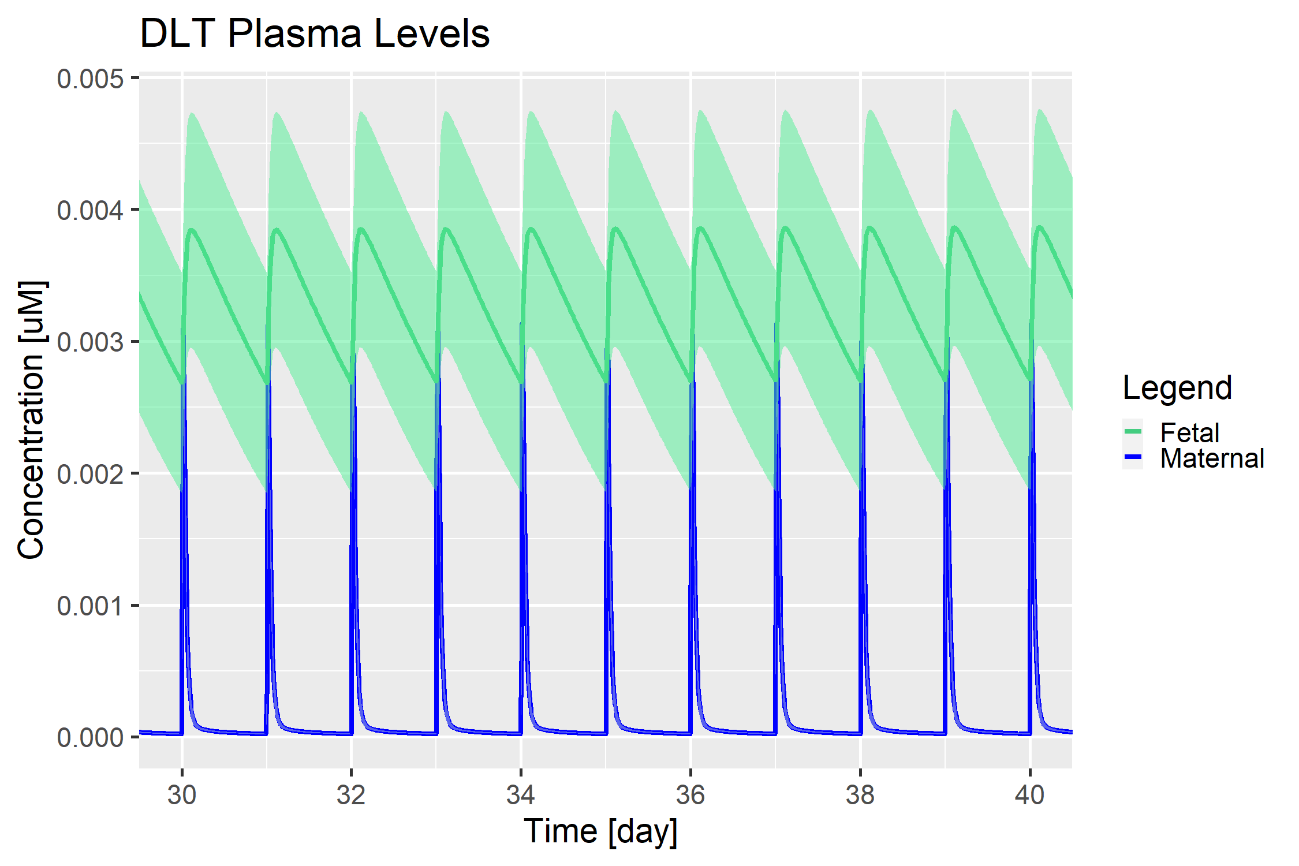


**Supp. Fig. 9:** Model simulations for Cases 3,5,6, and 7 after single daily administrations of 0.01 mg/kg (ADI) for maternal plasma (blue) and fetal plasma (green) zoomed into the last 10 days of simulation for better visualization. Solid lines represent the population means over 100 simulations, and shaded areas represent 1 standard deviation.

# Rat Model


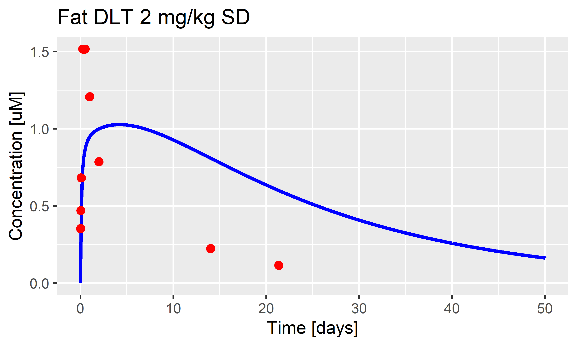

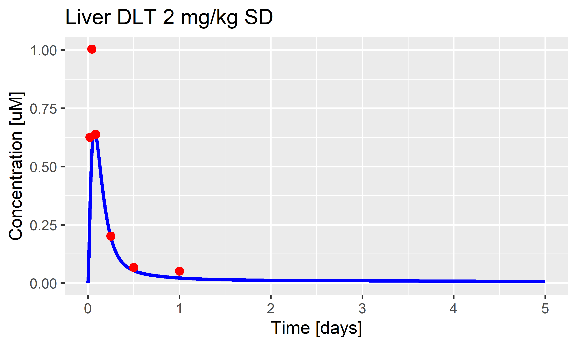

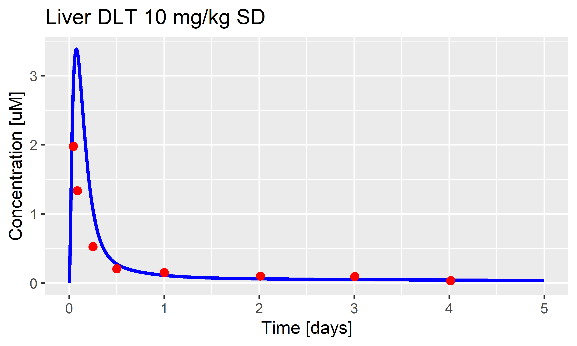

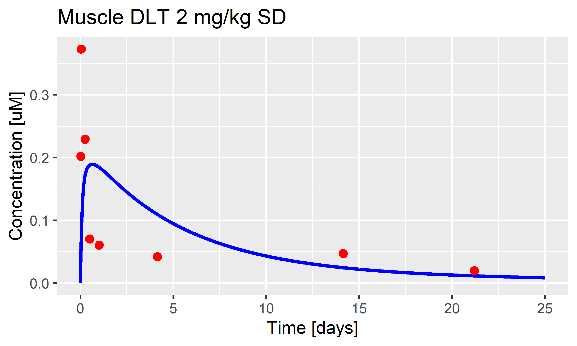

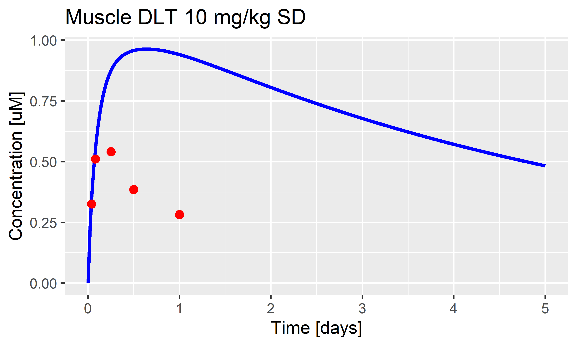

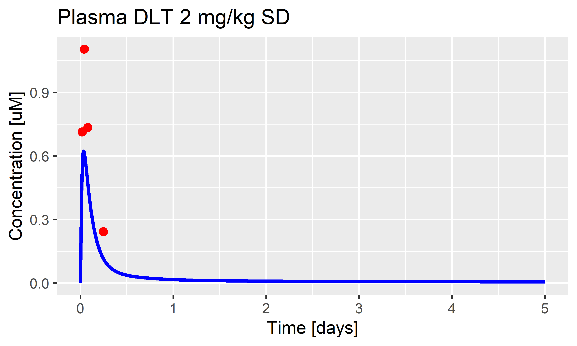


Supp. Fig. 10: Model simulations (solid blue lines) and PK measurements (red dots) after 2 and 10 mg/kg dosing in PND 90 rats (reference for experimentally measured data). The model captures the main trends in the observed data across a variety of doses. The maximum concentrations are over-and underpredicted across the investigated organs. However, deviations are within a factor of 2, which is considered acceptable in PBK modeling. Top left: adipose tissue 2 mg/kg, top right: liver 2 mg/kg, middle left: liver 10 mg/kg, middle right: muscle 2 mg/kg, bottom left: muscle 10 mg/kg, and bottom right: plasma 2 mg/kg.


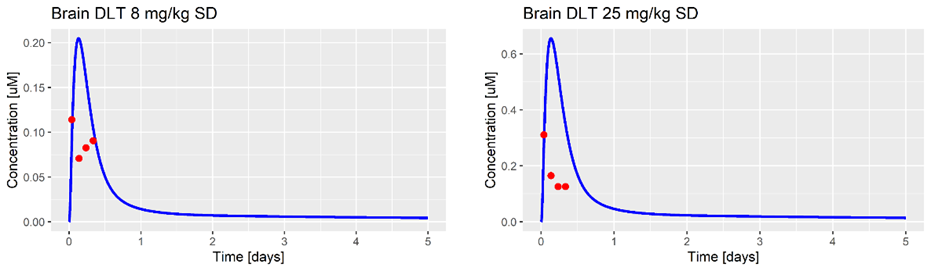


**Supp. Fig. 11:** The model was tested by trying to match PK data from experimental studies, where DLT was dissolved in corn oil after 8 mg/kg (left column) and 25 mg/kg (right column).

# REFERENCES

Abboud, T.K. *et al.* (1985) Comparative maternal and neonatal effects of halothane and enflurane for cesarean section. *Acta Anaesthesiol Scand*, **29**, 663–668.

Abboud, T.K. *et al.* (1983) Epidural bupivacaine, chloroprocaine, or lidocaine for cesarean section--maternal and neonatal effects. *Anesth Analg*, **62**, 914–919.

Aichhorn, W. *et al.* (2008) Olanzapine plasma concentration in a newborn. *J Psychopharmacol*, **22**, 923–924.

Ala-Kokko, T.I. *et al.* (1997) Feto-maternal distribution of ropivacaine and bupivacaine after epidural administration for cesarean section. *Int J Obstet Anesth*, **6**, 147–152.

Antunes, N. de J. *et al.* (2015) Influence of gestational diabetes on the stereoselective pharmacokinetics and placental distribution of metoprolol and its metabolites in parturients. *Br J Clin Pharmacol*, **79**, 605–616.

Arwood, L.L. *et al.* (1979) Placental transfer of theophylline: two case reports. *Pediatrics*, **63**, 844–846.

Aweeka, F.T. *et al.* (2010) Lopinavir protein binding in HIV-1-infected pregnant women. *HIV Med*, **11**, 232–238.

Bartels, P.A. *et al.* (2007) Nicardipine in pre-eclamptic patients: placental transfer and disposition in breast milk. *BJOG*, **114**, 230–233.

Bernard, B. *et al.* (1977) Maternal-fetal pharmacology of cefatrizine in the first 20 weeks of pregnancy. *Antimicrob Agents Chemother*, **12**, 231–236.

Best, B.M. *et al.* (2015) Pharmacokinetics of tenofovir during pregnancy and postpartum. *HIV Med*, **16**, 502–511.

Boulton, D.W. and Fawcett, J.P. (1997) Pharmacokinetics and pharmacodynamics of single oral doses of albuterol and its enantiomers in humans. *Clin Pharmacol Ther*, **62**, 138–144.

Bourget, P. *et al.* (1995) Disposition of a new rate-controlled formulation of prazosin in the treatment of hypertension during pregnancy: transplacental passage of prazosin. *Eur J Drug Metab Pharmacokinet*, **20**, 233–241.

Bourget, P. *et al.* (1991) Pharmacokinetics of tobramycin in pregnant women. Safety and efficacy of a once-daily dose regimen. *J Clin Pharm Ther*, **16**, 167–176.

Boyce, P.M. *et al.* (2011) Duloxetine transfer across the placenta during pregnancy and into milk during lactation. *Arch Womens Ment Health*, **14**, 169–172.

Buchanan, M.L. *et al.* (2009) Clonidine pharmacokinetics in pregnancy. *Drug Metab Dispos*, **37**, 702–705.

Caritis, S.N. *et al.* (2012) Relationship between 17-hydroxyprogesterone caproate concentrations and gestational age at delivery in twin gestation. *American Journal of Obstetrics & Gynecology*, **207**, 396.e1-396.e8.

Chappuy, H. *et al.* (2004) Maternal-fetal transfer and amniotic fluid accumulation of protease inhibitors in pregnant women who are infected with human immunodeficiency virus. *Am J Obstet Gynecol*, **191**, 558–562.

Choi-Kwon, S. *et al.* (2006) Fluoxetine treatment in poststroke depression, emotional incontinence, and anger proneness: a double-blind, placebo-controlled study. *Stroke*, **37**, 156–161.

Cigarini, I. *et al.* (1995) Epidural clonidine combined with bupivacaine for analgesia in labor. Effects on mother and neonate. *Reg Anesth*, **20**, 113–120.

Cimetidine in elective Caesarean section - Mccaughey - 1981 - Anaesthesia - Wiley Online Library.

Colbers, A.P.H. *et al.* (2013) The pharmacokinetics, safety and efficacy of tenofovir and emtricitabine in HIV-1-infected pregnant women. *AIDS*, **27**, 739–748.

Concheiro, M. *et al.* (2011) Simultaneous analysis of buprenorphine, methadone, cocaine, opiates and nicotine metabolites in sweat by liquid chromatography tandem mass spectrometry. *Anal Bioanal Chem*, **400**, 69–78.

Craft, I. *et al.* (1981) Placental Transfer of Cefuroxime. *BJOG: An International Journal of Obstetrics & Gynaecology*, **88**, 141–145.

Craft, I. and Forster, T.C. (1978) Materno-fetal cephradine transfer in pregnancy. *Antimicrob Agents Chemother*, **14**, 924–926.

Cressey, T.R. *et al.* (2012) Efavirenz pharmacokinetics during the third trimester of pregnancy and postpartum. *J Acquir Immune Defic Syndr*, **59**, 245–252.

Dailland, P. *et al.* (1989) Intravenous propofol during cesarean section: placental transfer, concentrations in breast milk, and neonatal effects. A preliminary study. *Anesthesiology*, **71**, 827–834.

Disposition and placental transfer of etidocaine in pregnancy - PubMed.

Ebara, H. *et al.* (1986) Digoxin- and digitoxin-like immunoreactive substances in amniotic fluid, cord blood, and serum of neonates. *Pediatr Res*, **20**, 28–31.

EFSA Panel on Contaminants in the Food Chain (CONTAM) *et al.* (2018) Risk for animal and human health related to the presence of dioxins and dioxin-like PCBs in feed and food. *EFSA Journal*, **16**, e05333.

Erkkola, R. *et al.* (1982) Transfer of propranolol and sotalol across the human placenta. Their effect on maternal and fetal plasma renin activity. *Acta Obstet Gynecol Scand*, **61**, 31–34.

Fox, G.S. and Houle, G.L. (1969) Transmission of lidocaine hydrochloride across the placenta during Caesarian section. *Can Anaesth Soc J*, **16**, 135–143.

Fujimoto, S. *et al.* (1986) Concentrations of ritodrine hydrochloride in maternal and fetal serum and amniotic fluid following intravenous administration in late pregnancy. *European Journal of Obstetrics & Gynecology and Reproductive Biology*, **23**, 145–152.

Fujimoto, S. *et al.* (1991) Levels of ritodrine hydrochloride in fetal blood and amniotic fluid following long-term continuous administration in late pregnancy. *European Journal of Obstetrics & Gynecology*, **38**, 15–18.

Gandar, R. *et al.* (1980) Serum level of ritodrine in man. *Eur J Clin Pharmacol*, **17**, 117–122.

Gepts, E. *et al.* (1986) Pharmacokinetics and placental transfer of intravenous and epidural alfentanil in parturient women. *Anesth Analg*, **65**, 1155–1160.

Gingelmaier, A. *et al.* (2006) Placental transfer and pharmacokinetics of lopinavir and other protease inhibitors in combination with nevirapine at delivery. *AIDS*, **20**, 1737–1743.

Gonçalves, P.V.B. *et al.* (2007) Determination of pindolol enantiomers in amniotic fluid and breast milk by high-performance liquid chromatography: applications to pharmacokinetics in pregnant and lactating women. *J Chromatogr B Analyt Technol Biomed Life Sci*, **852**, 640–645.

Gordon, A. *et al.* (2010) Buprenorphine transdermal system for opioid therapy in patients with chronic low back pain. *Pain Res Manag*, **15**, 169–178.

Gross, T.L. *et al.* (1985) Maternal and fetal plasma concentrations of ritodrine. *Obstet Gynecol*, **65**, 793–797.

de Haan, G.-J. *et al.* (2004) Gestation-induced changes in lamotrigine pharmacokinetics: a monotherapy study. *Neurology*, **63**, 571–573.

Haddad, J. *et al.* (1993) Oral acyclovir and recurrent genital herpes during late pregnancy. *Obstet Gynecol*, **82**, 102–104.

Heikkilä, A. and Erkkola, R. (1994) Review of β-Lactam Antibiotics in Pregnancy. *Clin. Pharmacokinet.*, **27**, 49–62.

Hendrick, V. *et al.* (2003) Placental passage of antidepressant medications. *Am J Psychiatry*, **160**, 993–996.

Hirt, D. *et al.* (2007) Pharmacokinetic modelling of the placental transfer of nelfinavir and its M8 metabolite: a population study using 75 maternal-cord plasma samples. *Br J Clin Pharmacol*, **64**, 634–644.

van Hoog, S. *et al.* (2012) Transplacental passage of nevirapine, nelfinavir and lopinavir. *Neth J Med*, **70**, 102–103.

Hurst, A.K. *et al.* (1998) Pharmacokinetic and pharmacodynamic evaluation of atenolol during and after pregnancy. *Pharmacotherapy*, **18**, 840–846.

Irestedt, L. *et al.* (1998) Pharmacokinetics and clinical effect during continuous epidural infusion with ropivacaine 2.5 mg/ml or bupivacaine 2.5 mg/ml for labour pain relief. *Acta Anaesthesiol Scand*, **42**, 890–896.

Kacirova, I. *et al.* (2010) Serum levels of lamotrigine during delivery in mothers and their infants. *Epilepsy Res*, **91**, 161–165.

Kacirova, I. *et al.* (2015) Serum levels of valproic acid during delivery in mothers and in umbilical cord - correlation with birth length and weight. *Biomedical Papers*, **159**, 569–575.

Kerenyi, T.D. *et al.* (1980) Transplancental cardioversion of intrauterine supraventricular tachycardia with digitalis. *Lancet*, **2**, 393–394.

Kim, D.K. *et al.* (2000) Serotonin transporter gene polymorphism and antidepressant response. *Neuroreport*, **11**, 215–219.

Kimberlin, D.F. *et al.* (1998) Pharmacokinetics of oral valacyclovir and acyclovir in late pregnancy. *American Journal of Obstetrics & Gynecology*, **179**, 846–851.

Kofahl, B. *et al.* (1993) Studies on placental transfer of celiprolol. *Eur J Clin Pharmacol*, **44**, 381–382.

Koristkova, B. *et al.* (2019) Lamotrigine drug interactions in combination therapy and the influence of therapeutic drug monitoring on clinical outcomes in paediatric patients. *Basic Clin Pharmacol Toxicol*, **125**, 26–33.

Kramer, W.B. *et al.* (1995) Placental transfer of sulindac and its active sulfide metabolite in humans. *Am J Obstet Gynecol*, **172**, 886–890.

Labovitz, E. and Spector, S. (1982) Placental theophylline transfer in pregnant asthmatics. *JAMA*, **247**, 786–788.

Lierde, M. van and Thomas, K. (1982) Ritodrine concentrations in maternal and fetal serum and amniotic fluid. **10**, 119–124.

Loughhead, A.M. *et al.* (2006) Placental passage of tricyclic antidepressants. *Biol Psychiatry*, **59**, 287–290.

Maberry, M.C. *et al.* (1992) Antibiotic concentration in maternal blood, cord blood and placental tissue in women with chorioamnionitis. *Gynecol Obstet Invest*, **33**, 185–186.

Mandelbrot, L. *et al.* (2001) Maternal-fetal transfer and amniotic fluid accumulation of lamivudine in human immunodeficiency virus-infected pregnant women. *Am J Obstet Gynecol*, **184**, 153–158.

McGowan, W.A. (1979) Safety of cimetidine in obstetric patients. *J R Soc Med*, **72**, 902–907.

Melander, A. *et al.* (1978) Transplacental passage of atenolol in man. *Eur J Clin Pharmacol*, **14**, 93–94.

Meuldermans, W. *et al.* (1986) Protein binding of the analgesics alfentanil and sufentanil in maternal and neonatal plasma. *Eur J Clin Pharmacol*, **30**, 217–219.

Michael, C.A. (1979) Use of labetalol in the treatment of severe hypertension during pregnancy. *Br J Clin Pharmacol*, **8**, 211S-215S.

Mirkin, B.L. (1971) Diphenylhydantoin: placental transport, fetal localization, neonatal metabolism, and possible teratogenic effects. *J Pediatr*, **78**, 329–337.

Mirochnick, M. *et al.* (2008) Lopinavir exposure with an increased dose during pregnancy. *J Acquir Immune Defic Syndr*, **49**, 485–491.

Moodley, D. *et al.* (2001) Pharmacokinetics of zidovudine and lamivudine in neonates following coadministration of oral doses every 12 hours. *J Clin Pharmacol*, **41**, 732–741.

Moore, R.G. and McBride, W.G. (1978) The disposition kinetics of diazepam in pregnant women at parturition. *Eur J Clin Pharmacol*, **13**, 275–284.

Morselli, P.L. *et al.* (1990) Placental transfer and perinatal pharmacokinetics of betaxolol. *Eur J Clin Pharmacol*, **38**, 477–483.

Myllynen, P. *et al.* (2001) Transplacental Passage of Oxcarbazepine and Its Metabolites In Vivo. *Epilepsia*, **42**, 1482–1485.

Myllynen, P.K. *et al.* (2003) Transplacental passage of lamotrigine in a human placental perfusion system in vitro and in maternal and cord blood in vivo. *Eur J Clin Pharmacol*, **58**, 677–682.

Nation, R.L. (1981) Meperidine binding in maternal and fetal plasma. *Clin Pharmacol Ther*, **29**, 472–479.

Nau, H. *et al.* (1980) Placental transfer and pharmacokinetics of primidone and its metabolites phenobarbital, PEMA and hydroxyphenobarbital in neonates and infants of epileptic mothers. *Eur J Clin Pharmacol*, **18**, 31–42.

Newport, D.J. *et al.* (2007) Atypical antipsychotic administration during late pregnancy: placental passage and obstetrical outcomes. *Am J Psychiatry*, **164**, 1214–1220.

Ohman, I. *et al.* (2000) Lamotrigine in pregnancy: pharmacokinetics during delivery, in the neonate, and during lactation. *Epilepsia*, **41**, 709–713.

Ohman, I. *et al.* (2005) Pharmacokinetics of gabapentin during delivery, in the neonatal period, and lactation: does a fetal accumulation occur during pregnancy? *Epilepsia*, **46**, 1621–1624.

Ohman, I. *et al.* (2002) Topiramate kinetics during delivery, lactation, and in the neonate: preliminary observations. *Epilepsia*, **43**, 1157–1160.

de Oliveira Baraldi, C. *et al.* (2011) Metformin pharmacokinetics in nondiabetic pregnant women with polycystic ovary syndrome. *Eur J Clin Pharmacol*, **67**, 1027–1033.

Onnen, I. *et al.* (1979) Placental transfer of atropine at the end of pregnancy. *Eur J Clin Pharmacol*, **15**, 443–446.

Petersen, M. *et al.* (1984) Disposition of betamethasone in parturient women after intramuscular administration. *British Journal of Clinical Pharmacology*, **18**, 383–392.

Pharmacokinetics and transplacental passage of imipenem during pregnancy - PubMed.

Pienimäki, P. *et al.* (1997) Pharmacokinetics of oxcarbazepine and carbamazepine in human placenta. *Epilepsia*, **38**, 309–316.

Pirhonen, J.P. *et al.* (1990) Single dose of nifedipine in normotensive pregnancy: nifedipine concentrations, hemodynamic responses, and uterine and fetal flow velocity waveforms. *Obstet Gynecol*, **76**, 807–811.

Rampono, J. *et al.* (2004) A pilot study of newer antidepressant concentrations in cord and maternal serum and possible effects in the neonate. *Int J Neuropsychopharmacol*, **7**, 329–334.

Rampono, J. *et al.* (2009) Placental transfer of SSRI and SNRI antidepressants and effects on the neonate. *Pharmacopsychiatry*, **42**, 95–100.

Reynolds, F. and Taylor, G. (1970) Maternal and neonatal blood concentrations of bupivacaine: a comparison with lignocaine during continuous extradural analgesia. *Anaesthesia*, **25**, 14–23.

Ripamonti, D. *et al.* (2007) Atazanavir plus low-dose ritonavir in pregnancy: pharmacokinetics and placental transfer. *AIDS*, **21**, 2409–2415.

Riva, E. *et al.* (1978) Pharmacokinetics of furosemide in gestosis of pregnancy. *Eur J Clin Pharmacol*, **14**, 361–366.

Roberts, I. *et al.* (1984) Paracetamol metabolites in the neonate following maternal overdose. *Br J Clin Pharmacol*, **18**, 201–206.

van Runnard Heimel, P.J. *et al.* (2005) The transplacental passage of prednisolone in pregnancies complicated by early-onset HELLP syndrome. *Placenta*, **26**, 842–845.

Rytting, E. *et al.* (2014) Pharmacokinetics of Indomethacin in Pregnancy. *Clin Pharmacokinet*, **53**, 545–551.

Saarikoski, S. (1976) Placental transfer and fetal uptake of 3H-digoxin in humans. *Br J Obstet Gynaecol*, **83**, 879–884.

Sethi, P. *et al.* (2019) Plasma Protein and Lipoprotein Binding of Cis- and Trans-Permethrin and Deltamethrin in Adult Humans and Rats. *Drug Metab Dispos*, **47**, 941–948.

Shnider, S.M. and Way, E.L. (1968) The kinetics of transfer of lidocaine (Xylocaine) across the human placenta. *Anesthesiology*, **29**, 944–950.

Silberschmidt, A.-L. *et al.* (2008) Nifedipine concentration in maternal and umbilical cord blood after nifedipine gastrointestinal therapeutic system for tocolysis. *BJOG: An International Journal of Obstetrics & Gynaecology*, **115**, 480–485.

Sioufi, A. *et al.* (1984) Oxprenolol placental transfer, plasma concentrations in newborns and passage into breast milk. *British Journal of Clinical Pharmacology*, **18**, 453–456.

Stek, A.M. *et al.* (2012) Effect of pregnancy on emtricitabine pharmacokinetics. *HIV Med*, **13**, 226–235.

Takaku, T. *et al.* (2015) Quantitative Structure–Activity Relationship Model for the Fetal–Maternal Blood Concentration Ratio of Chemicals in Humans. *Biological & Pharmaceutical Bulletin*, **38**, 930–934.

Takeda, A. *et al.* (1992) Protein binding of four antiepileptic drugs in maternal and umbilical cord serum. *Epilepsy Res*, **13**, 147–151.

Tomson, T. *et al.* (1997) Lamotrigine in pregnancy and lactation: a case report. *Epilepsia*, **38**, 1039–1041.

Trenque, T. *et al.* (1998) Human maternofoetal distribution of pyrimethamine-sulphadoxine. *Br J Clin Pharmacol*, **45**, 179–180.

Vanky, E. *et al.* (2005) Placental passage of metformin in women with polycystic ovary syndrome. *Fertil Steril*, **83**, 1575–1578.

Visser, A.A. and Hundt, H.K. (1984) The pharmacokinetics of a single intravenous dose of metronidazole in pregnant patients. *J Antimicrob Chemother*, **13**, 279–283.

Wang, X. *et al.* (2013) Quantitative determination of famotidine in human maternal plasma, umbilical cord plasma and urine using high-performance liquid chromatography-mass spectrometry. *Biomed Chromatogr*, **27**, 866–873.

Watts, D.H. *et al.* (1991) Pharmacokinetic Disposition of Zidovudine during Pregnancy. *The Journal of Infectious Diseases*, **163**, 226–232.

Wolff, F. *et al.* (1982) Perinatal pharmacokinetics of acetylsalicylic acid. *Arch. Gynecol.*, **233**, 15–22.

Young, O.M. *et al.* (2015) Pharmacokinetics of cefazolin prophylaxis in obese gravidae at time of cesarean delivery. *Am J Obstet Gynecol*, **213**, 541.e1–7.

Zastre, J. *et al.* (2013) Lack of P-Glycoprotein-Mediated Efflux and the Potential Involvement of an Influx Transport Process Contributing to the Intestinal Uptake of Deltamethrin, cis-Permethrin, and trans-Permethrin. *Toxicol Sci*, **136**, 284–293.
